# Facet‐Engineered Surface and Interface Design of Photocatalytic Materials

**DOI:** 10.1002/advs.201600216

**Published:** 2016-08-17

**Authors:** Song Bai, Lili Wang, Zhengquan Li, Yujie Xiong

**Affiliations:** ^1^ Hefei National Laboratory for Physical Sciences at the MicroscaleiChEM (Collaborative Innovation Center of Chemistry for Energy Materials)Hefei Science Center (CAS) and School of Chemistry and Materials ScienceUniversity of Science and Technology of ChinaHefeiAnhui230026China; ^2^ Key Laboratory of the Ministry of Education for Advanced Catalysis MaterialsCollege of Chemistry and Life SciencesInstitute of Physical and ChemistryZhejiang Normal UniversityJinhuaZhejiang321004China

**Keywords:** facets, surfaces, interfaces, photocatalysis, solar energy

## Abstract

The facet‐engineered surface and interface design for photocatalytic materials has been proven as a versatile approach to enhance their photocatalytic performance. This review article encompasses some recent advances in the facet engineering that has been performed to control the surface of mono‐component semiconductor systems and to design the surface and interface structures of multi‐component heterostructures toward photocatalytic applications. The review begins with some key points which should receive attention in the facet engineering on photocatalytic materials. We then discuss the synthetic approaches to achieve the facet control associated with the surface and interface design. In the following section, the facet‐engineered surface design on mono‐component photocatalytic materials is introduced, which forms a basis for the discussion on more complex systems. Subsequently, we elucidate the facet‐engineered surface and interface design of multi‐component photocatalytic materials. Finally, the existing challenges and future prospects are discussed.

## 1 Introduction

To alleviate the global energy crisis and environmental pollution, the photocatalysis that directly converts solar light into chemical energy has been widely explored over the past decades.^1^, ^2^, ^3^ As commonly recognized, the invention of photocatalysts with high activity, selectivity and stability is the prerequisite for putting “photocatalysis” into use in industry and in our lives.^4^, ^5^, ^6^ To achieve this goal, semiconductor materials with well‐defined sizes, structures, compositions and shapes have been extensively developed as photocatalysts for various reactions including water splitting, CO_2_ reduction and pollutant degradation.^7^, ^8^, ^9^ In particular, size shrinkage not only boosts the number of catalytic sites, but also may tailor the electronic structures of catalysts, which highlights the niches of photocatalytic materials at the microscale or nanoscale. The well‐defined parameters set up a platform for understanding their correlations with photocatalytic performance, allowing maneuvering the performance through parameter adjustment.^10^

During a typical photocatalytic process, charge kinetics plays a central role in the conversion of solar to chemical energy through generating and transferring charge carriers. In principle, three basic steps are involved in such a complex process in terms of charge kinetics: 1) charge generation under semiconductor photoexcitation; 2) charge transfer to catalyst surface; and 3) charge consumption in redox reactions on the catalyst surface.^11^ The efficiency of each step largely determines the overall performance of a photocatalyst. Thus it is imperative to facilitate and reconcile the steps for the improved solar‐to‐chemical energy conversion. In efforts to achieve this goal, the photocatalytic materials have been developed from bare semiconductors to multi‐component hybrid structures in recent years.^12^, ^13^ In the hybrid photocatalysts, the synergism between different components has been proven to extend light absorption range in charge generation, to reduce electron‐hole recombination in charge transfer, or to improve reactant adsorption and activation in charge consumption.^11^ The synergism relies on the surface and interface structures of hybrid photocatalysts, thereby holding the promise for optimizing photocatalytic performance.^13^, ^14^ The surface where the redox reactions take place has a huge impact on the efficiency of reaction molecules receiving the photogenerated charges toward product generation. This importance to surface reactions is generally applied to both bare semiconductors and hybrid structures. In the hybrid structures, an additional important parameter is the interface between two adjacent components where charge carriers are transferred. The structure and quality of interfaces hold the key to the performance of charge transfer from light‐harvesting component to reaction sites and between different light‐harvesting centers. Taken together, the structures of surface and interface should be tightly controlled to fully functionalize the multiple‐component material systems.

**Figure 1:**
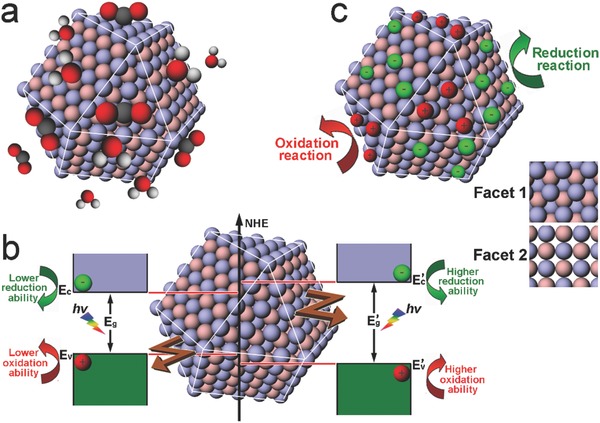
Schematics illustrating the important roles of facets in the surface design of photocatalytic materials. a) Adsorption and activation of reactant molecules on different facets. b) Redox abilities of photogenerated charge carriers tuned by the surface electronic band structures of different facets. c) Accumulations of photogenerated electrons or holes on different facets.

To control the surface and interface structures, the facets for forming the surface and interface are the key parameters that should be rationally selected. The facets exposed on photocatalyst surface may affect the photocatalytic performance through various working mechanisms. For instance, 1) surface atomic arrangements determine the adsorption and activation of reaction molecules, tuning catalytic activity and selectivity (**Figure** **1**a);^15^, ^16^ 2) the surface electronic band structures (i.e., surface states), which depend on surface facets, would provide the photogenerated charge carriers with tunable redox abilities for catalytic reactions (Figure 1b);^17^ 3) the efficiency of charge separation and transfer inside light‐harvesting semiconductors depends on crystal orientations, resulting in the varied charge densities for surface reactions.^18^, ^19^ Moreover, when the semiconductor is enclosed by multiple facets, the varied electronic band structures of surface facets may result in the spatial charge separation which accumulates the photogenerated electrons and holes on different facets for reduction and oxidation reaction, respectively (Figure 1c).^20^

**Figure 2:**
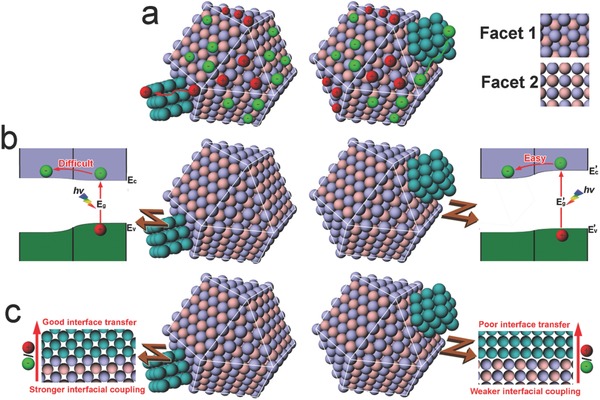
Schematics illustrating the important roles of facets in the interface design of photocatalytic hybrid materials. a) Interfacial charge transfer following the accumulation of photogenerated electrons or holes on different facets. b) Interfacial charge transfer efficiency depending on the component energy bands which are correlated their surface facets. c) Interfacial charge transfer relying on the structural and electronic couplings of components which vary with facet contacts.

As compared with bare semiconductors, the multi‐component systems are more complicated as the interfaces between the components become involved. The component facets that form the interfaces via solid‐solid contact may impact on interfacial charge transfer from several aspects. For instance, 1) interfacial charge transfer takes place after the charge carriers are transported from bulk to the interface, so the facet‐dependent charge accumulation as described above would maneuver the charge transfer (**Figure** **2**a);^20^ 2) the interfacial alignment of the energy bands that are strongly correlated with component facets determines the potential difference for driving interfacial charge transfer (Figure 2b);^21^ 3) the electronic coupling and defect density at the interface, depending on the compatibility of facet structures, largely affect the efficiency of charge transfer across the interface (Figure 2c).^22^ From the statements above, one can identify that highly efficient photocatalytic materials would be designed by tailoring the exposed facet on surface and the contact facets at interface.

To date, the advanced synthetic approaches to micro‐ and nanomaterials with well‐defined facets have significantly promoted the development of facet‐engineered surface and interface design in photocatalysis. The precisely controlled photocatalysts offer an ideal platform for investigating the dependence of photocatalytic performance on facets and the related mechanisms.^23^, ^24^, ^25^, ^26^ In this review, we focus on the recent advances in the facet‐engineered surface and interface design toward the photocatalysis applications. The review will begin with some key points which should receive attention in the facet‐engineered surface and interface design on photocatalytic materials. We will then discuss the synthetic approaches to realizing the facet control toward the surface and interface design of photocatalytic materials. In the following section, the facet‐engineered surface design on single‐component photocatalytic materials will be introduced. Subsequently, we will elucidate the facet‐engineered surface and interface design of multi‐component photocatalytic materials, respectively. Finally, the existing challenges and future prospects will be discussed.

## 2 Key Points to the Facet‐Engineered Surface and Interface Design

Engineering the facets at the locations of surface and interface is a challenging task in the design of photocatalytic materials. Prior to the following discussions, some key points to the facet‐engineered surface and interface design should be clarified.

1) The aim of this facet engineering is to enhance photocatalytic performance through rationally tailoring the surface and interface structures of photocatalysts. To reliably reflect the relationship between facets and photocatalytic performance, other parameters of the photocatalytic materials such as chemical compositions and crystal structures should be kept constant in control experiments. For this reason, the advanced synthetic protocols with high controllability over the parameters are required to exclude the interference from other parameter variations in the facet‐dependent studies.
2) The facet engineering on mono‐component photocatalytic materials (i.e., bare semiconductors) is relatively simple, as facet adjustment is only needed for the surface of light‐harvesting components. In comparison, the facet engineering on the photocatalytic hybrid structures is dramatically complicated as the involvement of multiple components leads to the increased number of surfaces as well as the formation of interface between the components. It should be noted that in some hybrid photocatalysts, certain surfaces do not serve as the catalytic active sites while some interfaces do not provide channels for charge carrier transportation. These possibilities make their facet adjustment unable to maneuver charge kinetics and to enhance photocatalytic performance. Thus the rational design on suitable surfaces and interfaces is highly important to the facet engineering.
3) In a hybrid structure, the structural correlation of surface with interface should be taken into account during the facet adjustment. For instance, when an interface is formed on the given component surface, the interfacial structure will inherit from the surface facet of this component. For this reason, controlling the exposed facet of an existing component inevitably results in variations in the facet structure of interface.

From the analysis above, one can envision that engineering the facets at the surface and interface of a hybrid structure would be a grand challenge. It not only calls for the advanced synthetic techniques with high precision (e.g., at the atomic level), but also requires high rationality for the intrinsic correlation of surface with interface and the relationship between structural characteristics and functionality.

## 3 Synthetic Approaches to Facet Control toward Surface and Interface Design

### 3.1 Facet Control on the Surface of Mono‐Component Photocatalytic Materials

For mono‐component structures, light‐harvesting semiconducors are the most widely used photocatalytic materials, and the facet engineering mainly focuses on their surface. Over the past decade, various synthetic approaches have been developed to control the exposed facets of photocatalytic semiconductor materials, including use of facet‐selective capping agents, kinetic control, thermodynamic control, and selective etching based on crystal anisotropy.

**Figure 3:**
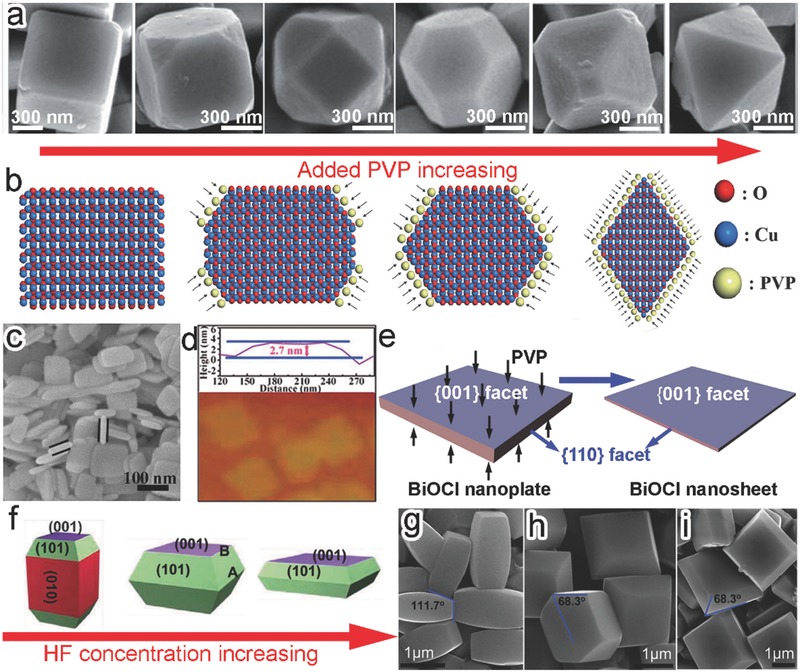
a) Scanning electron microscopy (SEM) images showing the evolution of Cu_2_O polyhedrons from cubes to octahedrons through increasing the amount of added PVP. b) Schematics illustrating the mechanism involved in the synthesis of Cu_2_O polyhedrons. Reproduced with permission.^29^ Copyright 2009, Royal Society of Chemistry. c) SEM image of the BiOCl nanoplates. d) Atomic force microscopy (AFM) image of ultrathin BiOCl nanosheets. e) Schematics illustrating the mechanism involved in the synthesis of 2D BiOCl nanostructures. Reproduced with permission.^30^ Copyright 2013, American Chemical Society. f–i) Schematic illustration (f) and (g–i) SEM images of anatase TiO_2_ crystals formed through increasing HF concentrations. Reproduced with permission.^32^

Capping agents have been extensively employed to manuever the facet growth of micro‐ and nanomaterials.^27^, ^28^ The capping agents can be selectively adsorbed to specific facets so as to suppress the growth along their axes. As a result, the facet with a slower growth rate will be more exposed on the surface. By increasing the amount of capping agent, the ratio of various facets can be tailored to ultimately leave the capped facet dominant on surface. For instance, the exposed facets of Cu_2_O were tailored by simply altering the amount of added poly(vinyl pyrrolidone) (PVP).^29^ In the absence of PVP, cubic Cu_2_O microcrystals enclosed with six {100} planes were obtained (**Figure** **3**a). The addition of PVP into the synthetic system resulted in the exposure of {111} surface as the preferential adsorption of PVP to the Cu_2_O{111} facets could hinder their growth (Figure 3b). The simultaneous {100} shrinkage and {111} enlargement on surface, enabled by the increase of PVP concentrations, led to the shape evolution from corner‐truncated cubes to cubooctahedrons and eventually to the highly symmetric octahedrons fully covered by {111} planes (Figure 3a,b).

Certainly the facet control by capping agents is not limited to the synthesis of symmetric polyhedral structures. This strategy has been validated for the formation of two‐dimensional (2D) photocatalytic materials that possess the high percentage of a single facet on their top and bottom flat surfaces. In a typical case, BiOCl nanoplates with thickness in ca. 30 nm and ultrathin nanosheets with thickness in ca. 2.7 nm were synthesized without and with PVP as capping agent, respectively (Figure 3c,d).^30^ The thickness shrinkage was attributed to the selective deposition of PVP on the top and bottom {001} planes, thereby preventing the axial growth (Figure 3e). As a result, the percentage of {001} planes increased from 62% in BiOCl nanoplates to 95% in ultrathin BiOCl nanosheets.

It should be noted that the polymeric capping agents would remain on the facets and influence the adsorption and activation of reactant molecules. This forms an obstacle for reliably evaluating the facet‐dependent photocatalytic performance. Small inorganic ions, alternative to organic surfactants and ligands, can be employed as the facet‐selective capping agents. This class of capping agents not only allows the access of reactants to the capped facets, but also can be more readily removed to obtain clean surface.^31^ Pan et al. employed F^–^ as a facet‐controlling agent to successfully tailor the exposed facets of anatase TiO_2_.^32^ As the F^–^ was selectively adsorbed to {001} planes, increasing the HF concentrations could inhibit the crystal growth along the [001] direction. As a result, the percentage of {001} planes on surface has been promoted from 14% to 24% and then to 40%, along with the shape evolution from columns to truncated bipyramids with different aspect ratios (Figure 3f–i). To the extreme, a further higher concentration of HF produced the TiO_2_ nanosheets dominated with {001} facet on the top and bottom surfaces as separately reported in another work.^33^

**Figure 4:**
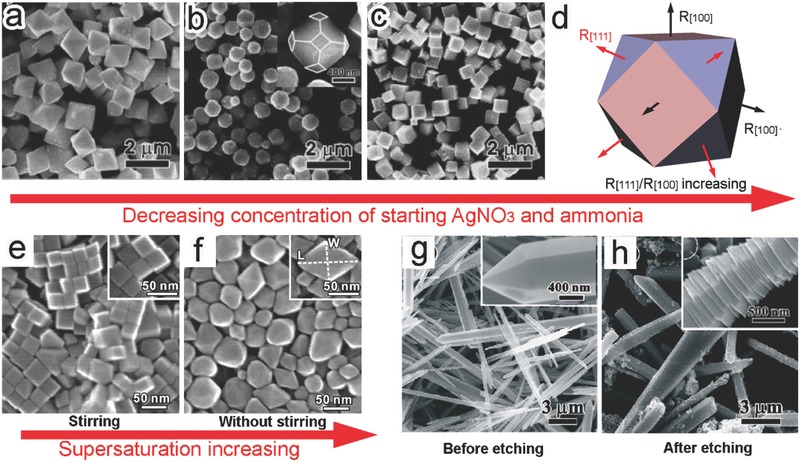
a–c) SEM images of Ag_2_O (a) octahedrons, (b) truncated octahedrons, and (c) cubes obtained through reducing the concentrations of starting materials. d) Schematic illustrating the corresponding synthetic mechanism. Reproduced with permission.^35^ Copyright 2010, American Chemical Society. e,f) SEM images of the (e) α‐Fe_2_O_3_ pseudocubes with {012} facets and (f) hexagonal bipyramidal α‐Fe_2_O_3_ with {113} facets prepared with and without solution stirring, respectively. Reproduced with permission.^37^ Copyright 2014, American Chemical Society. g,h) SEM images of ZnO columns (g) before and (h) after etching with OA. Reproduced with permission.^40^ Copyright 2010, American Chemical Society.

As demonstrated above, the facet control via capping agents can make the crystal growth deviated from the forms that are originally favored by thermodynamics.^34^ Kinetic control is another versatile approach to the thermodynamically disfavored surface facets.^23^ For instance, the exposed facets of Ag_2_O were tailored through altering the concentrations of starting materials.^35^ By lowering the concentrations of AgNO_3_ and NH_3_·H_2_O, the shapes of Ag_2_O crystals evolved from octahedrons bounded by {111} planes to truncated octahedrons by mixed {111}/{100} planes and then to cubes by {100} planes (**Figure** **4**a–c). In this case, the alteration of growth environments by reactant concentrations substantially manipulated the growth rates (R) of Ag_2_O along the [111] and [100] directions. At a sufficiently low reactant concentration, the ratio of R_[111]_ to R_[100]_ was increased to yield the cubic Ag_2_O crystals (Figure 4d).

In addition to kinetic control, the facets of semiconductor crystals can be maneuvered in the thermodynamic regime. As a matter of fact, the use of facet‐selective capping agents falls into the category of thermodynamic control: the selective adsorption of capping agents to specific facets reduces their surface energies.^34^ Nevertheless, the capping agents are not indispensible for the thermodynamic control. From the viewpoint of thermodynamics, a polyhedron enclosed by a single facet is preferentially formed when the surface energy of this facet is sufficiently low as compared with others. As the surface energy difference between the facets is minimized, more than one facet will appear on the surface of crystals. According to the Thomson‐Gibbs equation, the surface energy of a facet is in proportion to the supersaturation during crystal growth,^36^ providing a knob for tuning surface facets. For instance, the exposed facets of α‐Fe_2_O_3_ nanocrystals could be simply tailored by controlling the supersaturation.^37^ Stirring reaction solution resulted in the formation of α‐Fe_2_O_3_ pseudocubes enclosed with {012} facets in the synthetic system that originally yielded hexagonal bipyramidal α‐Fe_2_O_3_ nanocrystals with {113} facets (Figure 4e,f). The formation of {113} facets with higher surface energy was achieved by increasing the supersaturation without stirring.

It should be noted that in most cases, the facet engineering for photocatalytic semiconductors involves both thermodynamic and kinetic control which take facet stability and relative growth rate into account.^34^, ^38^ These methods mainly manipuated the assembly of atoms into crystals and thus represent the bottom‐up approach to facet engineering. In sharp contrast, the directional chemical etching based on crystal anisotropy is a widely used top‐down approach to engineer the surface facets of semiconductor materials.^23^, ^39^ In a typical example, the facet evolution from ZnO hexagonal columns enclosed with $\left\{\mathrm{10}\bar{1}0\right\}$ nonpolar faces to pagoda‐like ZnO bounded by $\left\{\mathrm{10}\bar{1}1\right\}$ and $\left\{\mathrm{000}\bar{1}\right\}$ polar planes has been achieved using oleic acid (OA) as a selective etching agent (Figure 4g,h).^40^ The reaction between Zn^2+^ cations and OA preferentially took place on the nonpolar $\left\{\mathrm{10}\bar{1}0\right\}$ faces composed of equivalent O^2–^ and Zn^2+^ ions. In comparison, the etching rates were relatively lower on the O‐terminated $\left\{\mathrm{10}\bar{1}1\right\}$ and $\left\{\mathrm{000}\bar{1}\right\}$ polar planes. During this process, the Zn^2+^ cations released from the etching would be nucleated and grown again to form hexagonal pyramids with stable polar facets.

### 3.2 Facet Control on the Surface of Multi‐Component Photocatalytic Materials

As photocatalytic materials are formed by combining multiple components, the facet adjustment for each component surface may benefit the optimization of photocatalytic performance as long as the surface participates in redox reactions. The complexity of hybrid structures increases the difficulty in simultaneously tailoring the exposed facets of components. Certainly this difficulty greatly depends on the combination methods for the components. The self‐assembly of two pre‐synthesized components offers the highest flexibility for facet engineering, as the facets of the components can be independently controlled in their own synthetic procedures.^41^ Another method is to in situ grow a new component on the surface of an existing one. In this synthetic system, the existing component serves as a seed and provides the growth sites in a seeding process. Similarly to the surface control of bare semiconductors, the facet of seeds can be facilely tailored by modifying the synthetic method.^21^, ^42^ However, it would be a challenge to maneuver the facet growth of the second component during the seeding process. In this section, the in situ synthetic approaches to surface facet control will be discussed according to architectural structures.

**Figure 5:**
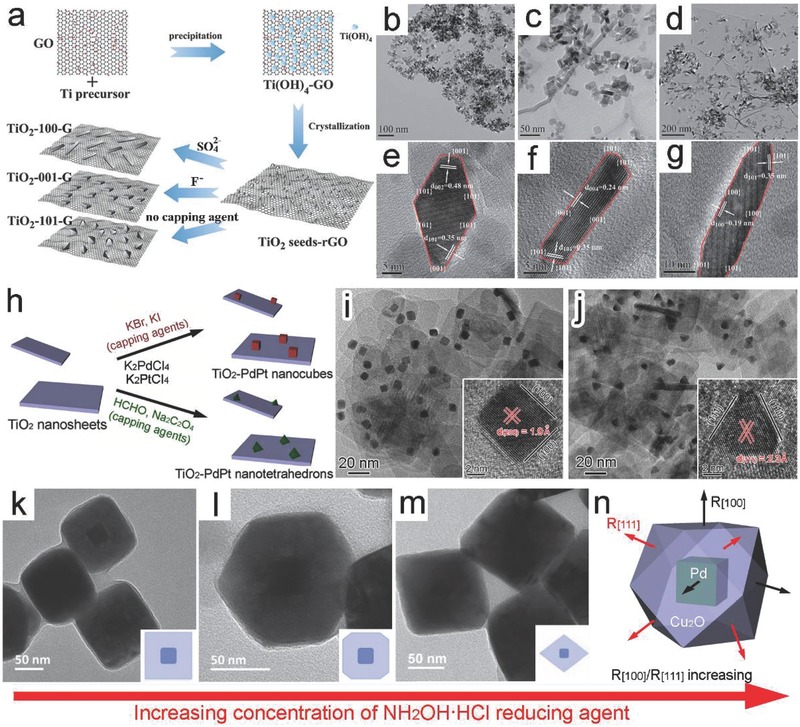
a) Schematic illustrating the synthesis of TiO_2_‐graphene nanocomposites with controllable TiO_2_ crystal facets. b–g) Transmission electron microscopy (TEM) and high‐resolution TEM (HRTEM) images of the as‐prepared (b,e) TiO_2_‐101‐G, (c,f) TiO_2_‐001‐G, and (d,g) TiO_2_‐100‐G. Reproduced with permission.^43^ h) Schematic illustration for the growth of PdPt cocatalysts enclosed with different facets on TiO_2_ nanosheets. i,j) TEM and HRTEM images of the as‐prepared (i) TiO_2_‐supported Pd_50_Pt_50_ nanocubes and (j) TiO_2_‐supported Pd_50_Pt_50_ nanotetrahedrons. Reproduced with permission.^44^ k–m) TEM images and scheme (inset) showing a gradual change in the shape of Cu_2_O shell from (k) cubes to (l) cuboctahedrons and then to (m) octahedrons in Pd‐Cu_2_O core‐shell structures through increasing the concentration of NH_2_OH·HCl. n) Schematic illustrating the corresponding synthetic mechanism. Reproduced with permission.^46^ Copyright 2013, Royal Society of Chemistry.

The supported structure is a widely used configuration for hybrid photocatalysts, in which the support is incompletely wetted by a newly formed component. To form this configuration, the exposed facet of new component is generally controlled by facet‐selective capping agents. For instance, capping agents have been employed to tailor the surface facets of TiO_2_ loaded on graphene nanosheets.^43^ To tailor the surface facets, different capping anions were used to guide the growth of crystalline TiO_2_ seeds into nanocrystals with graphene oxide and amorphous Ti(OH)_4_ as precursors (**Figure** **5**a).^43^ In the absence of capping agent, octahedral TiO_2_ nanocrystals enclosed by {101} facets (namely, TiO_2_‐101‐G) were formed. In comparison, TiO_2_ nanosheets dominated with {001} facets (TiO_2_‐001‐G) and TiO_2_ nanorods with {100} facets (TiO_2_‐100‐G) were formed on graphene when F^−^ and SO_4_^2−^ were used as capping agents, respectively (Figure 5b–g). Certainly the use of capping agents is not limited to the surface facet adjustment for light‐harvesting semiconductors. The metal component in a hybrid structure is commonly controlled through a similar strategy. A typical case is to tailor the exposed facets of PdPt alloy nanocrystals that were supported on TiO_2_ nanosheets.^44^ In the synthesis, Br^–^ and I^–^ ions facilitated the in situ growth of Pd_50_Pt_50_ nanocubes enclosed with {100} planes on TiO_2_ nanosheets, while the synergistic use of HCHO and Na_2_C_2_O_4_ as capping agents resulted in the coverage of {111} planes on Pd_50_Pt_50_ nanotetrahedrons (Figure 5h–j).

It is worth pointing out that controlling the exposed facet in a supported structure inevitably alters the interface facet between two components, as capping agents have their effects on the entire surface of the newly formed component. For instance, in the above TiO_2_‐PdPt hybrid structures, two different interfaces – TiO_2_{001}‐PdPt{100} and TiO_2_{001}‐PdPt{111} (Figure 5i,j) were formed when tailoring the surface facets of PdPt nanocrystals,^44^ which may induce distinct lattice mismatch at the interfaces. In contrast, the formation of core‐shell structures through an epitaxial growth offers an ideal platform for maintaining the quality of interfaces during the facet control. The exposed facet of the core can guide the epitaxial growth of shell component along crystal orientation to minimize the lattice mismatch. It has been revealed that the Au cores covered by {111} facets favor the formation of Au{111}‐Cu_2_O{111} interface, while the Au{100} facets provide growth sites for Au{100}‐Cu_2_O{100} interface.^45^ In the shell growth, thermodynamics or kinetics has to be tightly controlled for the rational facet adjustment. For instance, the exposed facets of Cu_2_O shells could be tailored by varying the amount of reducing agent during their epitaxial growth on Pd{100} cores.^46^ Increasing in the concentration of NH_2_OH**·**HCl, the shape of Cu_2_O gradually changed from cube to cuboctahedron and then to octahedron (Figure 5k–m). In other words, the exposed facets of Cu_2_O shells evolved from {100} to {111} while the Pd{100}‐Cu_2_O{100} interface was well maintained. This case perfectly highlights the importance of kinetic control: the growth rate of Cu_2_O in [111] direction (R_[111]_) is higher than that in [100] (R_[100]_) at low NH_2_OH·HCl concentration, and vice versa (Figure 5n).

### 3.3 Facet Control on the Interface of Multi‐Component Photocatalytic Materials

As claimed in Section 1, the interface of a hybrid structure largely determines the efficiency of photo‐induced charge carriers transferring through. Similarly to surface control, the difficulty for tailoring interfacial facets is dependent on the methods for integrating two components. During an epitaxial growth, the formation of a new component will minimize the lattice mismatch with the existing one toward the lowest interfacial energy,^45^, ^47^ reducing the flexibility of altering interfacial facets. Thus the interfacial facet adjustment is mainly accomplished in the supported structures through self‐assembly or non‐epitaxial growth. When both components are enclosed by or dominated with a single facet, the case is quite simple: the surface facets of two components determine the interfacial structure. However, when a component is covered by two or more facets, several different facet combination forms would be involved to complicate the case. In this section, we will mainly discuss how to control interfacial structures when a component is covered by multiple facets.

**Figure 6:**
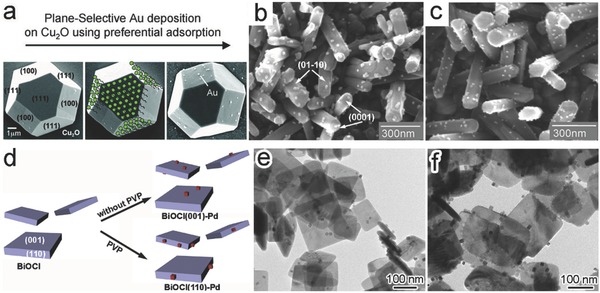
a) SEM images showing the facet‐selective Au deposition through the preferential adsorption of SDS to truncated octahedral Cu_2_O crystals. b,c) SEM images of the Au nanoparticles that were photochemically deposited on ZnO rods in (b) Au(CH_3_COO)_3_ and (c) AuCl_3_ ethanol solution, respectively. Reproduced with permission.^48^ Copyright 2009, American Chemical Society. d) Schematic illustration for the fabrication of different BiOCl‐Pd interfaces by employing PVP as a capping agent. e,f) TEM images of the obtained (e) BiOCl{001}‐Pd and (f) BiOCl{110}‐Pd hybrid structures. Reproduced with permission.^22^

In the interface control, the use of facet‐selective capping agents is still a widely used method. The capping agents not only affect facet growth, but also preclude the deposition of a new component on the capped facets. This preclusion would selectively form an interface between the new component and the uncapped facets. For instance, Au nanoparticles were selectively deposited on the {100} planes of truncated octahedral Cu_2_O crystals using sodium dodecyl sulfate (SDS) as a capping agent.^48^ The preferential adsorption of SDS to Cu_2_O{111} effectively blocked the nucleation of Au on the {111} planes, and as such, a Cu_2_O{100}‐Au interface was rationally formed (**Figure** **6**a). Another similar case is the selective photodeposition of Au on ZnO nanorods.^48^ Au nanoparticles were formed on both top {0001} and side $\left\{0\bar{1}\mathrm{10}\right\}$ planes with Au(CH_3_COO)_3_ as a precursor (Figure 6b). As AuCl_3_ solution was used as an alternative Au^3+^ source, Cl^–^ ions could be preferentially adsorbed on the polar {0001} planes to prevent the formation of ZnO{0001}‐Au interface (Figure 6c).

In the use of capping agents, one may take advantage of component sizes and shapes. A typical example is the selective formation of BiOCl‐Pd interfaces on the BiOCl nanoplates with large {001} surface (Figure 6d).^22^ Due to the high {001} surface coverage, Pd nanocubes were predominately assembled on the top and bottom {001} surfaces of BiOCl nanoplates to form a BiOCl{001}‐Pd{100} interface (Figure 6e). When the BiOCl nanoplates were firstly treated with PVP, the adsorption of PVP to BiOCl{001} could preclude the loading of Pd nanocubes on the BiOCl{001} facet. As a result, Pd nanocubes were exclusively attached to the four side {110} faces to form a BiOCl{110}‐Pd{100} interface (Figure 6f).

**Figure 7:**
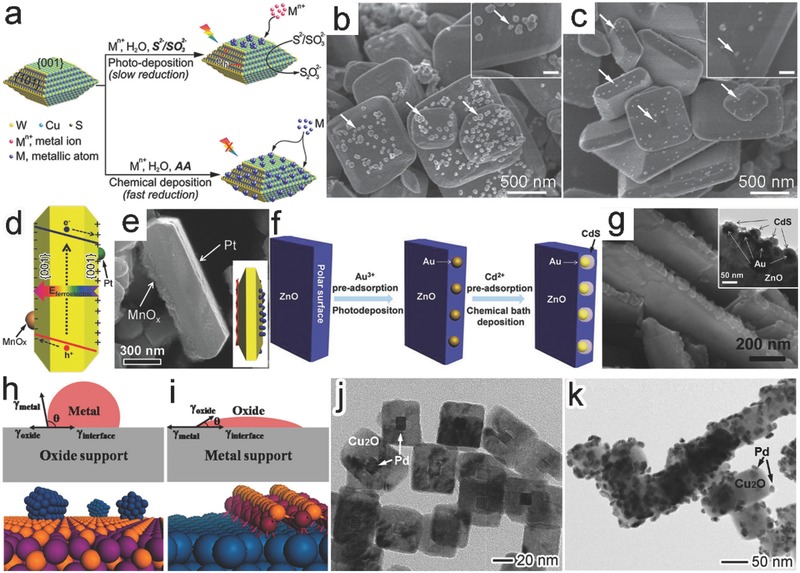
a) Schematic illustration for loading metal cocatalysts on a Cu_2_WS_4_ single crystal through photodeposition or chemical deposition. b,c) SEM images of 1 wt% Pt‐loaded Cu_2_WS_4_ photocatalysts prepared through (b) photoreduction and (c) chemical reduction, respectively. Reproduced with permission.^49^ d,e) Schematic illustration (d) and SEM image (e) for the selective deposition of MnO_x_ and Pt at the two sides of {001} facet on PbTiO_3_ nanoplates. Reproduced with permission.^52^ Copyright 2014, Royal Society of Chemistry. f) Schematic illustration for the selective deposition of Au/CdS induced by the surface polar charges of ZnO. g) SEM and TEM (inset) images of the prepared CdS/Au/ZnO structure. Reproduced with permission.^53^ Copyright 2013, Royal Society of Chemistry. h,j) Schematics for the metal‐oxide interface in the (h) oxide‐supported metal structure and (j) oxide‐on‐metal inverse structure (γ_oxide_ is the surface energy of oxide, γ_metal_ is the surface energy of metal, γ_interface_ is the interface free energy, and *θ* is the contact angle). Reproduced with permission.^57^ Copyright 2013, American Chemical Society. j,k) TEM images of Cu_2_O‐Pd hybrid structures obtained with j) Pd nanocubes and k) Cu_2_O nanocubes as existing components, respectively. Reproduced with permission.^58^

Given the detrimental effect of residual polymeric capping agents on photocatalysis, facet‐dependent photodeposition has been developed as an alternative route to facet‐selective interface formation. The adjacent facets of a semiconductor crystal may possess different band structures so that the photogenerated electrons and holes are separately accumulated on the different facets (i.e., spatial charge separation). This feature provides a driving force to selectively deposit the new components on the facets through reduction or oxidation reactions.^11^, ^20^ For instance, a photodeposition method has been employed to load metal cocatalysts on the surface of single‐crystal decahedral Cu_2_WS_4_, in comparison with the conventional chemical deposition (**Figure** **7**a).^49^ The conduction band (CB) and valence band (VB) edges of Cu_2_WS_4_ {101} facet are 80 and 60 meV higher than those of {001} facet, respectively, accumulating photoexcited electrons on the {001} facet. During the photodeposition, the reduction of PtCl_6_^–^ mainly occurred on the {001} facets of Cu_2_WS_4_ to form a Cu_2_WS_4_{001}‐Pt interface (Figure 7b). In contrast, Pt nanoparticles were formed through reduction by ascorbic acid (AA) and randomly deposited on both {101} and {001} facets of Cu_2_WS_4_ in a photo‐free chemical deposition system (Figure 7c).

The internal electric field built in ferroelectric materials or between polar facets can play a similar role in selective accumulation of electrons or holes. The field promotes the electron‐hole separation along a particular direction and accumulates electrons and holes on the two side facets perpendicular to the field direction.^11^, ^50^, ^51^ This charge accumulation provides the opportunity for selecting facets to form interfaces through photo‐deposition. For instance, an internal electric field is built in ferroelectric PbTiO_3_ nanoplates along the [001] direction, so the photogenerated electrons and holes diffuse to the positively and negatively charged {001} facets, respectively (Figure 7d).^52^ As such, when H_2_PtCl_6_ and MnSO_4_ were simultaneously photochemically reduced and oxidized, Pt and MnO_x_ would be asymmetrically deposited on the positively and negatively charged {001} facets of PbTiO_3_, respectively, forming two difference interfaces (Figure 7e).

The polar facets, which are enriched with opposite surface charges owing to their different termination patterns of bonding networks,^11^ may provide an alternative approach to the facet‐dependent photodeposition for interface design. Due to electrostatic interaction, the reactant anions or cations for component deposition are attracted to the positively or negatively charged facets, respectively, offering the facet‐dependent selective deposition. For instance, Au nanoparticles could be selectively deposited on the lateral plane of ZnO nanosheets, in which the Au‐based complex ions were attached to the negatively charged O‐terminated $\left\{\mathrm{000}\bar{1}\right\}$ facet and then photo‐reduced into Au nanoparticles (Figure 7f).^53^ Further with the Cd‐based complex ions, CdS was coated around the Au nanoparticles on the O‐$\left\{\mathrm{000}\bar{1}\right\}$ polar surface to form a CdS/Au/ZnO heterostructure (Figure 7f,g). Similarly the facet‐selective deposition of Pt on platelike WO_3_ was achieved through the dark sorption of [PtCl_6_]^2–^ on positively charged {020} facets. The efficiency of this selective deposition has been proven dependent on the pH value – a key parameter to electrostatic interaction.^54^

In terms of facet‐selective interface formation,^45^, ^55^, ^56^ the interfacial wetting of components is an important factor that has to be taken into account. For instance, as metals have larger surface energy than oxides, the metals can hardly wet the surface oxides according to Young's equation. On the contrary, wetting metal surface with oxides can be more readily achieved. For this reason, the surface facets of metals provide stronger guidance for the formation of interface with oxides (Figure 7h,i).^57^ For instance, in the synthesis of Cu_2_O‐Pd hybrid structures, the interface formation underwent different situations when Pd nanocubes and Cu_2_O nanocubes were used as starting components, respectively.^58^ The {100} planes of Pd nanocubes directed the formation of Pd{100}‐Cu_2_O{100} interface; however, the same interface could not be formed when the Cu_2_O nanocubes enclosed with {100} facet were used as the starting material (Figure 7j,k).

## 4 Facet Engineering for Mono‐Component Photocatalytic Materials

### 4.1 Large Percentage of Surface Facets with High Photocatalytic Activity

Section 3 has elucidated the synthetic approaches to engineering the facets at the surface and interface of photocatalytic materials. We are now in a position to discuss the design of high‐performance photocatalysts based on facet engineering. In the surface design of mono‐component semiconductor materials, the simplest way is to increase the percentage of the exposed facets with higher photocatalytic activity and selectivity. The large coverage of one facet on surface would facilitate the investigations on facet‐dependent photocatalytic performance and related mechanisms. Furthermore, the understanding gained from the mechanism research can provide a guidance for identifying the high‐activity facet for enhanced photocatalytic performance. This two‐way research mode has been widely used for the development of facet‐engineered photocatalysts.

**Figure 8:**
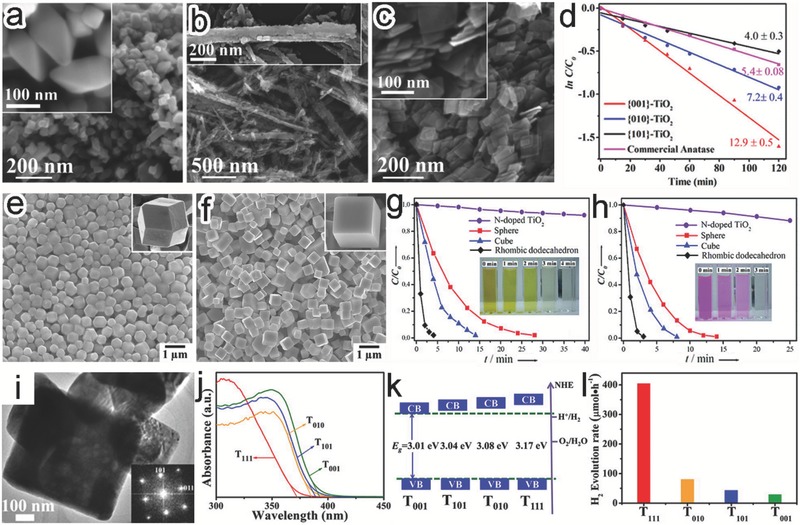
a–c) SEM images of (a) {101}‐TiO_2_, (b) {010}‐TiO_2_, and (c) {001}‐TiO_2_. d) pseudo‐first‐order plots for MO photodegradation using the differently faceted TiO_2_. Reproduced with permission.^60^ Copyright 2015, American Chemical Society. e–h) SEM images of Ag_3_PO_4_ (e) rhombic dodecahedrons and (f) cubes as well as their photocatalytic activities in (g) MO and (h) RhB degradation under visible‐light irradiation. Reproduced with permission.^61^ Copyright 2011, American Chemical Society. i) TEM image of TiO_2_ with {111} surface facets. j) UV‐visible absorption spectra of the TiO_2_ samples. k) Schematic illustration for the determined VB and CB edges of the TiO_2_ samples. l) Photocatalytic water splitting performance by Pt‐loaded (0.5%) TiO_2_ samples. Reproduced with permission.^17^ Copyright 2013, American Chemical Society.

The exposed facets may impact on photocatalytic performance through multiple effects. The most direct effect from surface facets is associated with the dangling bonds and low‐coordinated atoms at terraces, steps, kinks, adatoms, and vacancies. In addition to their different surface energies, these structural features affect the adsorption and activation of reactant molecules, varying photocatalytic activity and selectivity.^59^ For instance, anatase TiO_2_ nanooctahedrons (**Figure** **8**a), nanobelts (Figure 8b), and nanoplates (Figure 8c) were dominated with or enclosed by {101}, {010} and {001} facets, respectively. Their performance comparison revealed that {001}‐TiO_2_ provided 1.79 and 3.22 times higher reaction rates than {010}‐ and {101}‐TiO_2_ in photocatalytic degradation of methyl orange (MO), respectively (Figure 8d),^60^ as the {001} facet possessed higher surface energy and a larger number of coordinatively unsaturated Ti^4+^ active sites. In another case, the Ag_3_PO_4_ rhombic dodecahedrons enclosed by twelve {110} facets (Figure 8e) exhibited superior photocatalytic activity to the cubes bounded by six {100} facets (Figure 8f) in degradation of MO (Figure 8g) and rhodamine B (RhB) (Figure 8h).^61^ The density functional theory (DFT) calculation revealed that the surface energy of {110} facets (1.31 J/m^2^) was higher than that of {100} facets (1.12 J/m^2^), indicating the higher reactivity of {110} facets.

From the viewpoint of charge kinetics, the surface facets of semiconductors may possess different electronic band structures, caused by their atomic arrangements. The shift of CB and VB energy levels by surface states would directly alter the reduction and oxidation potentials of photogenerated carriers, respectively.^11^, ^32^ This feature has been recognized in the photocatalytic activity comparison between square‐shaped TiO_2_ plate covered by {111} facet (T_111_, Figure 8i) and TiO_2_ mainly enclosed with {001}, {101} or {010} facets (named as T_001_, T_101_ or T_010_).^17^ Ultraviolet (UV)‐visible absorption spectroscopy revealed that the bandgaps of T_001_, T_101_, T_010_ and T_111_ were 3.01, 3.04, 3.08 and 3.17 eV, respectively (Figure 8j). Despite their comparable VB maxima (VBMs), the four samples have CB minima (CBMs) in the order of T_111_ > T_010_ > T_101_ > T_001_ (Figure 8k). As a higher CBM can offer the electrons with higher potential for photocatalytic reduction reaction, the photocatalytic H_2_ evolution rate by the samples followed the same order as CBMs (T_111_ > T_010_ > T_101_ > T_001_) (Figure 8l).

**Figure 9:**
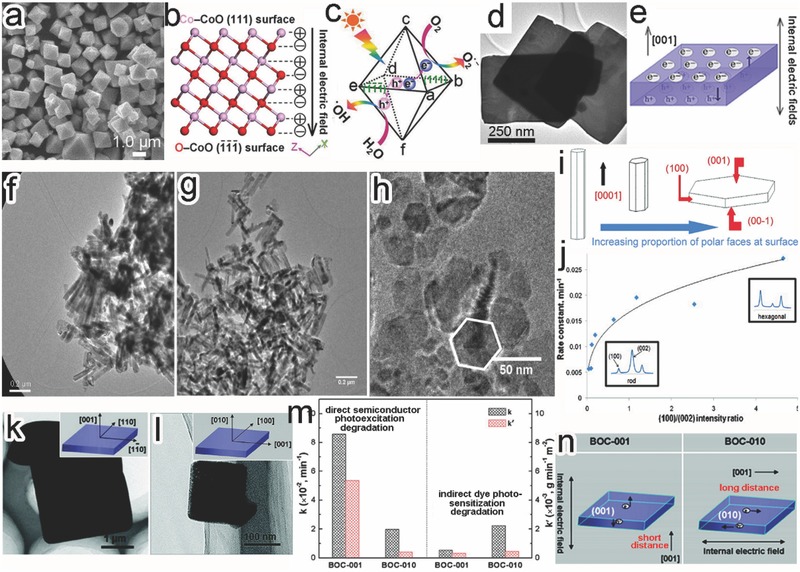
a) SEM image of CoO octahedrons. b) Atomic charge distribution of CoO {111} and $\left\{\bar{1}\bar{1}\bar{1}\right\}$ facets. c) Schematic illustration for the charge separation between polar {111} surfaces. Reproduced with permission.^62^ Copyright 2015, American Chemical Society. d) TEM image of Bi_4_Ti_3_O_12_ nanosheets dominated with {001} facets. e) Schematic illustration for the charge separation along [110] direction. Reproduced with permission.^63^ Copyright 2014, Elsevier. f–h) TEM images of (f) long ZnO rods, (g) short ZnO rods, and (h) hexagonal ZnO plates. i) Schematic diagrams showing the increased proportion of polar facets from rods to plates. j) Plot of the rate constants *vs*. (100)/(002) intensity ratio in MB photodegradation. Reproduced with permission.^64^ Copyright 2009, American Chemical Society. k,l) TEM images and scheme (inset) of (k) BOC‐001 and (l) BOC‐010. m) Comparison of reaction rate constants between BOC‐001 and BOC‐010 in MO degradation under (left) UV and (right) visible‐light irradiation. n) Schematic for the corresponding mechanism. Reproduced with permission.^18^ Copyright 2012, American Chemical Society.

Another important effect from surface facets is the dependence of charge transfer and separation on crystal orientation from two angles, intrinsically driven by internal electric field. Firstly, polar facets may spontaneously induce a polarization effect.^11^, ^50^ In the CoO octahedrons enclosed with polar {111} plane (**Figure** **9**a), internal electric field was established through the spontaneous polarization between the alternate layers of positive Co^2+^ ions and negative O^2–^ ions along the [111] direction (Figure 9b).^62^ The electric field could drive the migration of photogenerated electrons toward the positive polar Co‐CoO{111}, $\left\{\bar{1}\mathrm{11}\right\}$, $\left\{\bar{1}\bar{1}1\right\}$ and $\left\{1\bar{1}1\right\}$ surfaces for reduction reaction, along with the hole transfer to negative polar O‐CoO$\left\{\bar{1}\bar{1}\bar{1}\right\}$, $\left\{1\bar{1}\bar{1}\right\}$, $\left\{\mathrm{11}\bar{1}\right\}$ and $\left\{\bar{1}1\bar{1}\right\}$ for oxidation reaction (Figure 9c). Secondly, a spontaneous polarization takes place in ferroelectric materials as their positive and negative charges have different centers of symmetry.^11^, ^50^, ^51^ For instance, ferroelectric Bi_4_Ti_3_O_12_ nanosheets were dominant with {001} facets (Figure 9d),^63^ and exhibited a spontaneous polarization of 4 μC cm^–2^ along the c‐axis. The internal electric field drove the separation of the photogenerated electrons and holes and their diffusion along [001] direction (Figure 9e). The electrons and holes would be eventually accumulated on the two sides of the {001} facet, resulting in the superb photocatalytic activity of this facet.

In a similar case, the ZnO long nanorods, short nanorods and hexagonal plates, with the increased proportion of {001} polar faces, were used as photocatalysts in degradation of methylene blue (MB) (Figure 9f–i).^64^ The reaction rates turned out to promote with the increase of {001} proportion, suggesting that the terminal polar {001} and $\left\{\mathrm{00}\bar{1}\right\}$ facets were more active in the reaction (Figure 9j). Certainly the shape evolution from ZnO nanorods to nanoplates reduced the distance along the [001] direction for charge transfer and separation as well, contributing to the performance enhancement. Similarly, the BiOCl nanosheets enclosed with {001} facets (BOC‐001) showed higher photocatalytic activity in both the direct semiconductor photoexcitation degradation and indirect dye photosensitization degradation of MO, as compared with BiOCl nanosheets dominated with {010} facets (BOC‐010) (Figure 9k‐m).^18^ In this system, an internal electric field assisted the charge separation and transfer along the [001] direction in the BiOCl crystal structures, designating the direction of charge kinetics. As such, the BOC‐001 became more favorable in charge separation and transfer as its smaller dimension in the [001] direction substantially shortened the diffusion distance of photoinduced charge carriers (Figure 9n).

Overall, increasing the percentage of high activity facets is a versatile approach to develop highly efficient photocatalysts, which calls for the fundamental research on facet‐dependent performance assessement and mechanism investigation. In general, two major schemes have widely used in the pursuit of covering a semiconductor with a large portion of highly reactive facets. One is to develop symmetric polyhedral micro/nanocrystals enclosed by the high‐activity facet, such as the aforementioned Ag_3_PO_4_ rhombic dodecahedron enclosed by {110} facet (Figure 8e) and CoO octahedron enclosed by polar {111} facet (Figure 9a).^61^, ^62^ However, symmetric polyhedral structures have been rarely reported for few semiconductors which mainly possess a body‐centered cubic or face‐centered cubic crystal structure. Thus the other scheme that two‐dimensional micro/nanostructures have high‐activity facets on their flat surfaces has been more extensively explored in recent years. The typical examples include the TiO_2_ nanosheets with top and bottom planes of {001} facets (Figure 8c), TiO_2_ plates with majority {111} facets (Figure 8i), and Bi_4_Ti_3_O_12_ nanosheets (Figure 9d) and ZnO nanoplates (Figure 9h) dominated with polar {001} facets.^17^, ^60^, ^63^, ^64^

**Table** **1** summarizes the facet‐dependent activities of semiconductors in various photocatalytic reactions and the corresponding mechanisms, together with the photocatalyst shapes with high activity. It clearly shows that the activity of a facet is strongly dependent on the type of photocatalytic reactions and the related catalytic mechanisms. For example, the activity of anatase TiO_2_ facets in degradation of dyes are in the order: {001}>{010}>{101},^60^ while a different order ({010}>{101}>{001}) has been resolved for photocatalytic H_2_ evolution.^17^, ^32^ This feature manifests that the species adsorption and activation plays a more prominent role in dye degradation, while the water splitting raises higher demand for band structures.^85^ In many cases, the activity of a facet is influenced by a mix of different catalytic mechanisms. For instance, both internal electronic field and species adsorption contributed to the superior photocatalytic activity of BiOBr{001} to BiOBr{010} in degradation of 2,4‐dichlorophenol and inactivation of *Escherichia coli*;^74^, ^75^ CeO_2_{100} and CeO_2_{110} offered suitable band structure for generating more energetic holes and more O vacancies for catalytic active sites, respectively, which caused to exhibit different photoreactivitiy in photocatalytic oxidation of volatile organic compound and O_2_ evolution reaction.^79^

**Table 1:** Facet‐dependent activity of semiconductors in various photocatalytic reactions as well as their corresponding mechanisms

Semiconductor	Crystal phase	Facet activity comparison	Photocatalytic reaction	Mechanism	Shape of semiconductor (dominated/enclosed with high activity facet)	Ref.
TiO_2_	Anatase	{001}>{010}>{101}	Degradation of MO	Adsorption and activation	Sheet (Dominated)	60
Ag_3_PO_4_	Body‐centered cubic	{110}>{100}	Degradation of MO and RhB	Adsorption and activation	Rhombic dodecahedron (Enclosed)	61
TiO_2_	Anatase	{111}>{010}>{101}>{001}	H_2_ evolution from water	Band structures/adsorption and activation	Plate (Dominated)	17
ZnO	Hexagonal wurtzite	{001}>{100}	Degradation of MB	Direction of charge transfer and separation	Hexagonal plate (Dominated)	64
BiOCl	Tetragonal	{001}>{010}	Degradation of MO	Direction of charge transfer and separation	Plate (Dominated)	18
Cu_2_O	Face‐centered cubic	{111}>{100}	Degradation of MO	Adsorption and activation	Octahedron (Enclosed)	29, 65
TiO_2_	Anatase	{010}>{101}>{001}	Hydroxyl radicals production\H_2_ evolution from water	Band structures/adsorption and activation	Cuboid (Dominated)	32, 66
TiO_2_	Anatase	{010}>{101}>{001}	Reduction of CO_2_	Adsorption and activation	Rod (Dominated)	67
Cu_2_O	Face‐centered cubic	{110}>{100}	Degradation of MO	Adsorption and activation	Rhombic dodecahedra (Enclosed)	68
AgBr	Face‐centered cubic	{111}>{100}	Degradation of MO	Adsorption and activation	Octahedron (Enclosed)	69
Ag_2_O	Face‐centered cubic	{100}>{110}>{111}	Degradation of MO	Adsorption and activation	Cube (Enclosed)	70, 71
BiOI	Tetragonal	{110}>{001}	Degradation of bisphenol	Adsorption and activation/band structures	Sheet (Dominated)	72
Ag_3_PO_4_	Body‐centered cubic	{111}>{100}>{110}	O_2_ evolution from water	Adsorption and activation/direction of charge transfer and separation	Tetrahedron (Enclosed)	73
BiOBr	Tetragonal	{001}>{010}	Degradation of 2,4‐dichlorophenol	Adsorption and activation/direction of charge transfer and separation	Sheet (Dominated)	74
			Inactivation of Escherichia coli			75
CdS	Hexagonal	{0001}>$\left\{1\bar{0}11\right\}$	H_2_ evolution from water	Adsorption and activation/band structures	Plate (Dominated)	76
Ag_2_WO_4_	Orthorhombic	{110}>{011}>{010}>$\left\{0\bar{1}0\right\}$	Degradation of RhB and Rhodamine 6G (Rh6G) dyes	Adsorption and activation	Hexagonal rod (Dominated)	77
BiOCl	Tetragonal	{010}>{001}	Degradation of 2‐naphthol	Adsorption and activation	Sheet (Dominated)	78
CeO_2_	Cubic fluorite	{100}>{110}	Oxidation of volatile organic compounds	Band structures	Cube (Enclosed)	79
		{110}>{100}	O_2_ evolution from water	Adsorption and activation	Rod (Dominated)	
SrTiO_3_	Cubic	{101}>{100}	Degradation of RhB and MO	Adsorption and activation	Triangular prisms (Dominated)	80
SrTiO_3_	Cubic	{100}>{110}	Decomposition of acetic acid	Direction of charge transfer and separation	Cube (Enclosed)	81
		{110}>{100}	H_2_ evolution from water		Flake (Dominated)	
AgCl	Face‐centered cubic	{100}>{111}	Degradation of MO and RhB	Band structures	Cube (Enclosed)	82
AgCl	Face‐centered cubic	{15 5 2}>{311}>{111}	Degradation of MO	Adsorption and activation	Concave hexoctahedron (Enclosed)	83
BiOBr	Tetragonal	{102}>{001}	Degradation of RhB	Band structures	Sheet (Dominated)	84

### 4.2 Synergism between Different Surface Facets with Optimal Ratios

**Figure 10:**
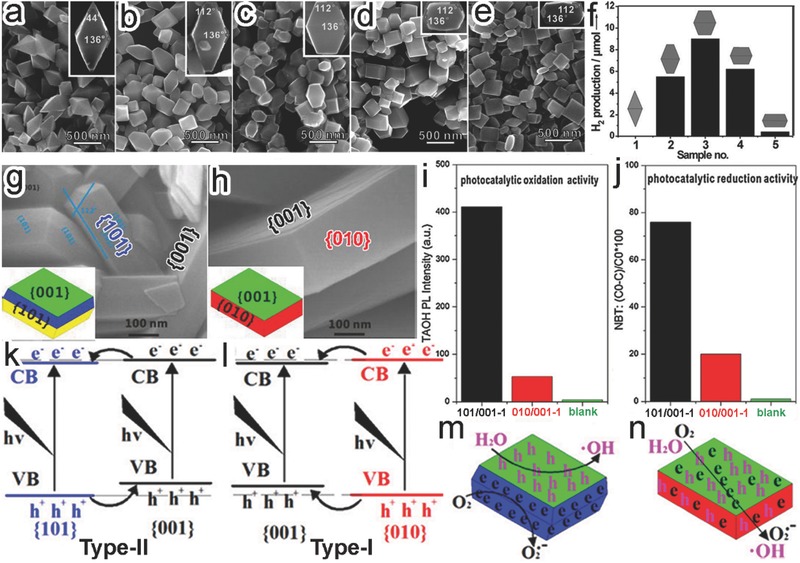
a–e) SEM images of TiO_2_ nanocrystals with the increased ratios of exposed {001} to {101} facets. f) Photocatalytic hydrogen production by the TiO_2_ with different exposed facets. Reproduced with permission.^86^ g,h) SEM images and scheme (inset) of (g) {101}/{001} and (h) {010}/{001} TiO_2_ samples. i,j) Comparison of photocatalytic (i) oxidation and (j) reduction activity by the {101}/{001} and {010}/{001} samples. k–n) Electronic band structures and charge distribution of the (k,m) type‐II {101}/{001} and (l,n) type‐I {010}/{001} samples. Reproduced with permission.^87^ Copyright 2013, Elsevier.

According to the analysis above, it is anticipated that a high proportion of high‐activity facet on surface would promote the performance of photocatalytic materials. However, in some cases, a synergism of different facets on the surface of semiconductor crystals may also further enhance their photocatalytic activity. As discussed above, the electrons and holes are separately accumulated on the different facets of a semiconductor, driven by their different band structures, thereby reducing the adverse electron‐hole recombination. Moreover, spatially separating reduction from oxidiation sites by different facets may also prevent the back reaction between reduction and oxidation products. The work by Liu et al. provided a very straightforward proof for the synergism of different facets in achieving the enhanced photocatalytic activity.^86^ They compared the photocatalytic H_2_ production by a group of anatase TiO_2_ nanocrystals with tunable percentages of {001} facet area from 0 to 51.2% (**Figure** **10**a–e). The truncated tetragonal bipyramidal TiO_2_ sample with 14.9% {001} facets turned out to achieve the highest photocatalytic activity, well exceeding those by the octahedral TiO_2_ fully enclosed by {101} facets or the sample with highest {001} percentage (51.2%) (Figure 10f). In the case of mixed {101} and {001} facets, the photogenerated electrons and holes migrated toward {101} and {001} facets, respectively, balancing their recombination and reaction rates.

It should be noted that only when the electronic structures of two facets are satisfied with the Type‐II (staggered) band alignment (i.e., one facet has higher CBM and VBM than the other one), can the spatial charge separation be achieved. In a typical case, anatase TiO_2_ crystals with {101}/{001} (Figure 10g) and {010}/{001} (Figure 10h) coexistence facets were synthesized by Ye et al., respectively.^87^ The {101}/{001} TiO_2_ possessed significantly higher photocatalytic activity than {010}/{001} TiO_2_ in both oxidation and reduction reactions (Figure 10i,j) owing to their unique Type‐II band alignment. Both the CBM and VBM of {001} facet were higher than those of {101} in the {101}/{001} TiO_2_, forming a Type‐II band alignment. As such, the photoinduced electrons and holes would transfer to {101} and {001} facets, respectively, which accomplished the spatial charge separation between the two facets (Figure 10k). When {101} facets were replaced by {010}, a Type‐I (straddling) band alignment would be formed as the CBM and VBM of {010} facets were higher and lower than those of {001}, respectively. In this case, most electrons and holes would transfer to the {001} facets together and would be no longer separated (Figure 10l). As a result, the photocatalytic reduction and oxidation by {101}/{001} TiO_2_ should take place on {001} and {101} facets, respectively, whereas the two half reactions could not be spatially separated in {010}/{001} TiO_2_ (Figure 10 m,n). As summaried in **Table** **2**, thus far the spatial charge separation between different facets has been validated for various semiconductor micro/nanocrystals covered by two or more facets.

**Table 2:** Spatial charge separation between different facets in various photocatalytic semiconductors

Semiconductor	Crystal phase	Shape of semiconductor	Facet for reduction reaction	Facet for oxidation reaction	Ref.
TiO_2_	Anatase	Tetragonal bipyramid	{101}	{001}	86, 87, 88, 89, 90, 91, 92
BiVO_4_	Monoclinic	Tetragonal bipyramid	{010}	{110}	20, 93, 94, 95
BiOCl	Tetragonal	Sheet	{001}	{110}	22, 96
CeO_2_	Body‐centered cubic	Hexahedron prism‐anchored octahedron	{111}	{100}	97
Cu_2_O	Face‐centered cubic	Cuboctahedron	{100}	{111}	21
Cu_2_WS_4_	Decahedral	Tetragonal bipyramid	{001}	{101}	49, 98
TiO_2_	Brookite	Sheet	{210}/{101}	{201}	99
TiO_2_	Rutile	Tetragonal prism	{110}	{011}	100
TiO_2_	Anatase	Cuboid	{101}	{001}/{100}	101
TiO_2_	Anatase	Truncated tetragonal bipyramid	{101}	{001}/{110}	102
SrTiO_3_	Cubic	Tetrahexahedron	{001}	{023}	103, 104
BaLa_4_Ti_4_O_15_	Perovskite	Sheet	{111}	{110}	105, 106
Cu_2_O	Face‐centered cubic	26‐facet polyhedral cube	{100}	{111}	107
Cu_2_O	Face‐centered cubic	Concave cubooctahedron	{104}	{100}	108

**Figure 11:**
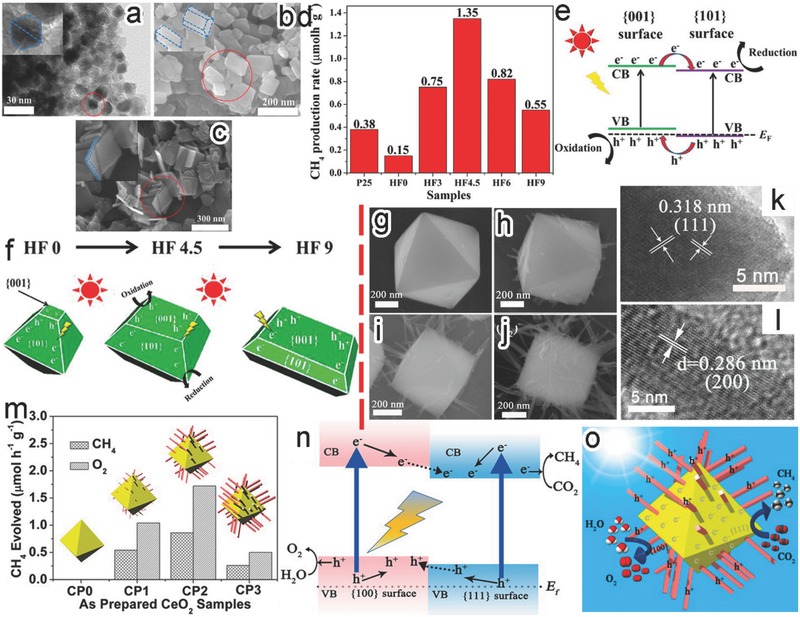
a–c) TEM and SEM images of TiO_2_ nanocrystals with the increased ratios of exposed {001} to {101} by varying HF amount: (a) HF0, (b) HF4.5, and (c) HF9. d) Photocatalytic CH_4_ production activity by the TiO_2_ samples. e) Electronic band structures for {001} and {101} facets. f) Schematic illustration for the spatial separation on the HF0, HF4.5, and HF9 samples designating redox sites. Reproduced with permission.^88^ Copyright 2014, American Chemical Society. g–j) SEM images of (g) CeO_2_ octahedrons and (h–j) hexahedron prism‐anchored CeO_2_ octahedrons with the increased portions of hexahedron prisms. k,l) HRTEM images for the (k) octahedron and (l) prism in the hexahedron prism‐anchored octahedronal CeO_2_, respectively. m) CH_4_ and O_2_ generation rates by the samples of redox sites with 0.5% wt Pt. n,o) Schematic illustration for (n) the spatial charge separation between {001} and {111} facet and (o) the photocatalytic mechanism of the CeO_2_ homojunction. Reproduced with permission.^97^ Copyright 2015, American Chemical Society.

To maximize the synergistic effect between different semiconductor facets, one can optimize the exposed area ratio of the synergism facets. For instance, the photocatalytic CO_2_ reduction activity by anatase TiO_2_ was enhanced by exposing both {001} and {101} facets on TiO_2_ surface.^88^ Adjusting the amounts of HF, the ratio of the exposed TiO_2_ {101} to {001} facets could be tuned from 89:11 (i.e., {101}‐dominated octahedral bipyramid, HF0) to 17:83 (i.e., {001}‐dominated nanoplates, HF9) (**Figure** **11**a–c). With photocatalytic CO_2_ reduction as a model reaction, it revealed that the optimal facet ratio was 45:55 (i.e., HF4.5) for achieving the highest CH_4_ production (Figure 11d). As a Type II band alignment was formed between {101} and {001} facets, photogenerated electrons and holes would migrate toward {101} and {001} facets, respectively, during the photocatalytic process (Figure 11e). As illustrated in Figure 11f, most of the photogenerated electrons and holes in the HF0 were mainly accumulated on the {101} facets due to the low percentages of {001} facet, inducing serious charge carrier recombination. The similar situation happend for the HF9 in which the charge carriers were accumulated and recombined on the {001} facet. Only in the optimal ratio of {101} to {001} facets (i.e., 45:55, HF4.5), the most efficient spatial charge separation could be achieved for reduction and oxidation reactions.

As limited by the crystal structures and synthetic techniques, not all the combinations of two facets can be achieved in a single‐domain crystal. Thus the artificial synthesis of crystal facet‐based homojunctions offers an alternative approach to achieve the synergism between two facets through spatial charge separation. An excellent demonstration is the CeO_2_ homojunction consisting of hexahedron prism‐anchored octahedron fabricated through crystallographic‐oriented epitaxial growth.^97^ The hexahedron prism‐anchored CeO_2_ octahedrons and bare CeO_2_ octahedrons were selectively obtained by controlling the addition of phosphate ions (Figure 11g,h). Increasing the concentrations of phosphate ions, the prism arms became denser, longer and thicker (Figure 11h–j). As shown in HRTEM images, the CeO_2_ octahedrons and hexahedron prisms were enclosed with {111} and {100} facets, respectively (Figure 11k,l). When the samples were used in photocatalytic reduction of CO_2_, no hydrocarbon species product was found for bare CeO_2_ octahedrons while hexahedron prism‐anchored octahedrons exhibited distinct photocatalytic activity in CH_4_ generation (Figure 11m). With the increase of hexahedron prism arms, the CH_4_ generation rates displayed a volcano trend, suggesting the synergistic effect between {111} and {100} facets. An optimal {111}/{100} ratio ensured to obtain the highest photocatalytic activity. Figure 11n shows the Type II band alignment between {111} and {100} facets, in which the photogenerated electrons and holes transferred to the CB of {111} and the VB of {100} facets, respectively. Driven by this spatial charge separation, the electrons were accumulated on {111} facets to perform the reduction of CO_2_ to CH_4_, and the holes were gathered on {100} facets for H_2_O oxidation (Figure 11o).

## 5 Facet Engineering for Multi‐Component Photocatalytic Materials

### 5.1 Facet‐Engineered Surface Design

In a multi‐component hybrid structure, junctions are mainly formed through the combination of a semiconductor with other materials (e.g., semiconductors, metals, and carbon materials) to form different charge kinetic models.^11^, ^14^, ^109^ To promote the performance of a component participating in surface catalytic reactions, the surface facet of this component should be tightly controlled. Meanwhile, the facet adjustment would inevitably result in variations in its interfacial structure with other components in contact. This factor should certainly be considered; otherwise, interfacial charge transfer would become the bottleneck of the entire charge kinetics in photocatalysis so as to reduce the efficacy of surface design.

A hybrid structure involves multiple components including the light‐harvesting semiconductor and other materials, all for which the surface facet design can be performed. The case of semiconductor component is relatively straightforward: the designing rules in Section 4 can be directly implemented. For instance, the graphene‐supported TiO_2_ nanosheets dominated with {001} facet have been widely used as photocatalysts for dye degradation mainly owing to the high activity of TiO_2_{001} facet;^110^, ^111^, ^112^ the Ag_3_PO_4_ tetrahedrons were integrated with C_3_N_4_ nanosheets for photocatalytic MB degradation by taking advantage of the highest activity of Ag_3_PO_4_{111} facet.^113^

**Figure 12:**
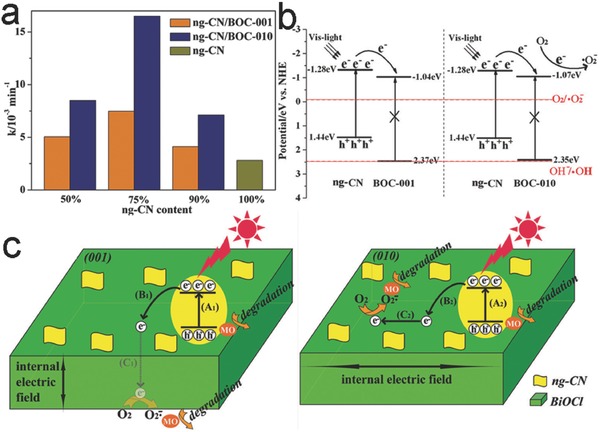
a) Visible‐light‐induced photocatalytic degradation of MO over bare ng‐CN, ng‐CN/BOC‐001 and ng‐CN/BOC‐010 heterojunction photocatalysts containing different proportions of ng‐CN. b) Band alignments in ng‐CN/BOC‐001 and ng‐CN/BOC‐010 heterojunction photocatalysts. c) Proposed mechanism for photocatalytic reactions occurring on ng‐CN/BOC‐001 and ng‐CN/BOC‐010 heterojunction photocatalysts. Reproduced with permission.^114^ Copyright 2015, Royal Society of Chemistry.

Nevertheless, we have to point out that the selection of a high‐activity semiconductor facet does not necessarily guarantee the high photocatalytic activity of semiconductor‐based hybrid structure. As mentioned in Section 4, the UV‐excitable BiOCl nanosheets dominated with {001} facets (BOC‐001) possessed higher photocatalytic activity than BOC‐010, owing to the internal electric field along [001] direction.^18^ However, the situation totally changed when the g‐C_3_N_4_ nanoparticles – a visible‐light semiconductor sensitizer – were loaded on BOC‐001 and BOC‐010 nanosheets. The ng‐CN/BOC‐010 heterojunction photocatalysts exhibited superior photocatalytic performance to ng‐CN/BOC‐001 in MO degradation under visible‐light irradiation (**Figure** **12**a). Although the photogenerated electrons could effectively migrate from the CB of n‐C_3_N_4_ to the CB of BiOCl in both composites (Figure 12b), driven by the [001]‐orientated internal electric field in BiOCl, the traveling lengths for electron transfer were quite different. The electrons in ng‐CN/BOC‐001 were required to travel within the BOC‐001 bulk, while the electron transfer in ng‐CN/BOC‐010 occurred along the BOC‐010 surface. Apparently, the BOC‐010 offered a shorter distance for charge transport, lowering the loss of electrons during their delivery to reduction reaction sites (Figure 12c).^114^

Similarly, Pt loading can alter the activity order of anatase TiO_2_{001} and {010} facets for the photocatalytic reduction of CO_2_ to CH_4_.^115^ In the absence of Pt loading, TiO_2_{010} facet exhibited higher activity in photocatalytic CO_2_ reduction in comparison with TiO_2_{001}, attributing to the stronger CO_2_ adsorption and longer charge lifetime of TiO_2_{010}. Further integrated with small Pt nanoparticles, however, the TiO_2_{001} sample offered more efficient electron‐hole separation than the Pt‐TiO_2_{010} junction, enabling higher photocatalytic activity. This case further emphasizes that the significant impact of interface on charge transfer should not be neglected when the surface facets of photocatalytic hybrid structures are tailored.

**Figure 13:**
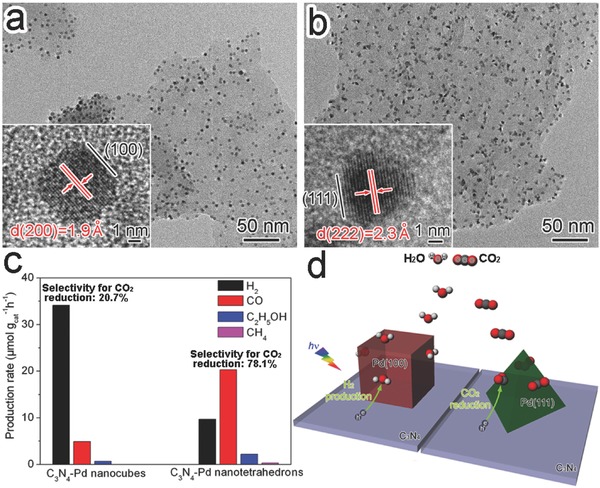
a,b) TEM and HRTEM (inset) images of (a) C_3_N_4_‐Pd nanocubes enclosed with Pd{100} facets and (b) C_3_N_4_‐Pd nanotetrahedrons with Pd{111} facets. c) Photocatalytic generation rates of H_2_ and carbon products by the catalysts with ca. 6 wt% Pd loading. d) Schematic illustration for the photocatalytic CO_2_ reduction in presence of H_2_O on C_3_N_4_‐Pd hybrid structures. Reproduced with permission.^118^ Copyright 2014, Royal Society of Chemistry.

In terms of Pt loading, the cocatalysts – Pt nanoparticles – not only trap the electrons to promote charge separation, but also provide active sites for CO_2_ adsorption and activation. As such, photocatalytic performance is no longer dependent on the activity of TiO_2_ facets.^115^, ^116^ Given the role of cocatalysts as reactant adsorption and activation sites, it would be straightforward to tune the photocatalytic performance by tailoring the surface facets of cocatalysts while maintaining the exposed facet of light‐harvesting semiconductor.^117^ In a typical case, we have tuned the selectivity of C_3_N_4_‐Pd hybrid structures in photocatalytic CO_2_ reduction in present of H_2_O through adjusting the surface facets of Pd cocatalysts.^118^ When the {100}‐faceted Pd nanocubes were supported on the g‐C_3_N_4_ nanosheets, the photocatalyst preferred to undergo the reduction of H_2_O to H_2_. In contrast, with the Pd nanotetrahedrons enclosed with {111} facets as cocatalyst, photocatalytic reduction mainly took place along the reduction of CO_2_ to carbon products (e.g., CO, C_2_H_5_OH, and CH_4_) (**Figure** **13**a,b). The selectivity for CO_2_ reduction was 20.7% of C_3_N_4_‐Pd nanocubes *versus* 78.1% of C_3_N_4_‐Pd nanotetrahedrons (Figure 13c), simply because the Pd{111} and Pd{100} facets offered higher adsorption and activation abilities for CO_2_ and H_2_O, respectively (Figure 13d). It should be pointed out that ensuring the equivalent efficiency of interfacial charge transfer is a prerequisite for reliably assessing the facet‐dependent reaction selectivity. In the case of g‐C_3_N_4_, this prerequisite was ensured by the unique conjugated structure of C_3_N_4_ which offered multiple coupling configurations with Pd facets in C_3_N_4_‐Pd{100} and C_3_N_4_‐Pd{111}. In addition to Pd, the surface facets of Pt and PdPt alloy cocatalysts have been reported to play an important role in the surface H_2_O adsorption and activation for photocatalytic H_2_ evolution.^44^, ^119^

### 5.2 Facet‐Engineered Interface Design

As briefly mentioned above, facet‐engineered interface design is a highly important theme to photocatalytic hybrid materials. As the interface is formed by integrating two components in contact, the design of interfacial facets can be carried out through tailoring the exposed facet of either semiconductor or other components (or a second semiconductor).^43^, ^120^ However, the variation in the component surface facets may also alter the process of surface reactions, complicating the correlation of photocatalytic performance with interfacial facets. As discussed in Section 3.3, it is a more straightforward strategy for simplifying the case by selectively depositing a new component on the different facets of an existing one.^20^, ^49^ On the whole, the main mission for facet‐engineered interface design is to select suitable component facets for forming the interface toward efficient charge transfer.

In general, there are multiple factors affecting interfacial charge transfer efficiency. The first factor is the ability of accepting photogenerated electrons or holes correlated with the facets of semiconductor. This correlation is caused by the spatial charge separation between adjacent facets or the internal electronic field along a particular crystal orientation as discussed above. For this reason, a suitable facet of semiconductor should be selected for the formation of interface with cocatalysts. As the cocatalysts trap electrons (or holes) and provide reactive sites for reduction (or oxidation) reactions, the selective deposition of reduction (or oxidation) cocatalysts on the electron (or hole)‐accumulated facets should be favorable to the overall photocatalytic performance. By rationally engineering the interfacial facets, the role of cocatalysts in charge trapping further promotes the spatial charge separation between different facets and prevents the back reactions of products. Furthermore, the accumulation of charge carriers on the facets facilitates the electron/hole transfer across semiconductor‐cocatalyst interface, allowing them to arrive at the surface of cocatalysts for reduction/oxidation reactions.^11^

**Figure 14:**
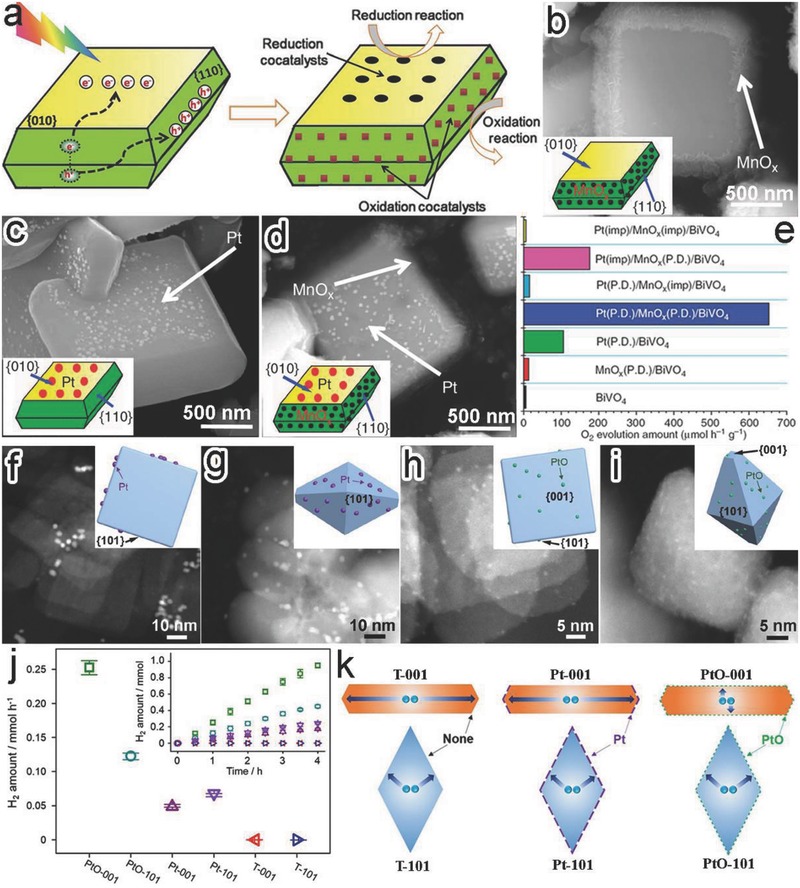
a) Schematic for selective deposition of reduction and oxidation cocatalysts on the {010} and {110} facets of BiVO_4_ based on the charge separation between different facets. b–d) SEM images and schematic illustration (inset) for (b) MnO_x_(P.D.)/BiVO_4_, (c) Pt(P.D.)/BiVO_4_, and (d) Pt(P.D.)/MnO_x_(P.D.)/BiVO_4_ hybrid structures. e) Photocatalytic water oxidation performance of BiVO_4_ based structures. Reproduced with permission.^20^, ^93^ Copyright 2013, Nature Publishing Group and Copyright 2014, Royal Society of Chemistry. f–i) TEM images and geometric models (inset) of (f) Pt‐001, (g) Pt‐101, (h) PtO‐001 and (i) PtO‐101. j) H_2_ evolution rates by bare TiO_2_ and TiO_2_ loaded with PtO clusters and metallic Pt cocatalyst, respectively. k) Schematic illustrating the migration of the photogenerated electrons in TiO_2_‐based photocatalysts. Reproduced with permission.^121^ Copyright 2015, Elsevier.

Here we employ the selective deposition of cocatalysts at the reduction and oxidation facets of monoclinic BiVO_4_ crystal as a typical example to demonstrate the importance.^20^, ^93^ Owing to the facet‐dependent band structures, the spatial charge separation of BiVO_4_ accumulated electrons and holes on {010} and {110} facet, respectively. The charge accumulation provided the charge‐enriched sites for the selective photodeposition of Pt (reduction) and MnO_x_ (oxidation) cocatalysts (**Figure** **14**a). Taking advantage of the accumulated electrons and holes, BiVO_4_{010}‐Pt and BiVO_4_{110}‐MnO_x_ interfaces (namely, Pt(P.D.)/BiVO_4_ and MnO_x_(P.D.)/BiVO_4_ hybrid structures) were selectively formed through the photo‐reduction of PtCl_6_^2–^ and photo‐oxidation of Mn^2+^, respectively (Figure 14b,c). Certainly the two interfaces could be integrated in a single hybrid structure Pt(P.D.)/MnO_x_(P.D.)/BiVO_4_ through the simultaneous photo‐reduction of PtCl_6_^2–^ and oxidation of Mn^2+^ (Figure 14d). As anticipated, the Pt(P.D.)/MnO_x_(P.D.)/BiVO_4_ achieved the highest photocatalytic oxygen evolution activity among the samples (Figure 14e). This manifests the synergism of BiVO_4_{010}‐Pt and BiVO_4_{110}‐MnO_x_ interfaces in boosting the photocatalytic performance. In particular, the performance well exceeded those by the hybrid structures such as Pt(imp)/MnO_x_(imp)/BiVO_4_, Pt(imp)/MnO_x_(P.D.)/BiVO_4_ and Pt(P.D.)/MnO_x_(imp)/BiVO_4_ in which co‐catalysts were randomly deposited on BiVO_4_ through an impregnation method (Figure 14e). It again demonstrates that high photocatalytic performance requires to deposit reduction and oxidation cocatalysts on the designated semiconductor facets.

It is worth mentioning that the facet‐dependent charge accumulation relies on the migration distance of charge carriers to surface facets. In certain geometric shapes, the charge carriers may travel along significantly different lengths to various facets inside a semiconductor. A short migration distance can suppress electron‐hole recombination and facilitate charge accumulation at the facet, so interfacial charge transfer would be enhanced by selectively depositing a new component on the semiconductor facet with short charge traveling length. For instance, Pt and PtO reduction cocatalysts were deposited on the TiO_2_ nanosheets (T‐001) and octahedrons (T‐101) that were dominated with {001} and {101} facets, respectively, to form four different interfaces (namely, Pt‐001, Pt‐101, PtO‐001 and PtO‐101).^121^ As indicated by TEM analysis, the metallic Pt cocatalyst was preferentially anchored on the TiO_2_{101} facet, while the PtO could be stabilized on both {001} and {101} facets (Figure 14f–i). During the photocatalysis, the Pt‐101 exhibited higher activity than the Pt‐001 in H_2_ evolution, while the activity of PtO‐001 exceeded that of PtO‐101 (Figure 14j). In this system, the Pt‐101 and PtO‐001 shortened the electron migration path as compared with the Pt‐001 and PtO‐101, respectively (Figure 14k); the distance between the center of semiconductor and cocatalysts was ∼2 nm in PtO‐001, ∼25 nm in Pt‐001, and ∼12 nm in both PtO‐101 and Pt‐101. Although TiO_2_{101} was the reduction facet with electron accumulation by spatial charge separation (Figure 14k),^86^, ^87^, ^88^, ^89^, ^90^, ^91^, ^92^ the Pt‐101 with Pt selectively deposited on {101} exhibited the lowest photocatalytic activity. This case well demonstrates that both spatial charge separation and charge migration length should be taken into account in the interface facet design.

**Figure 15:**
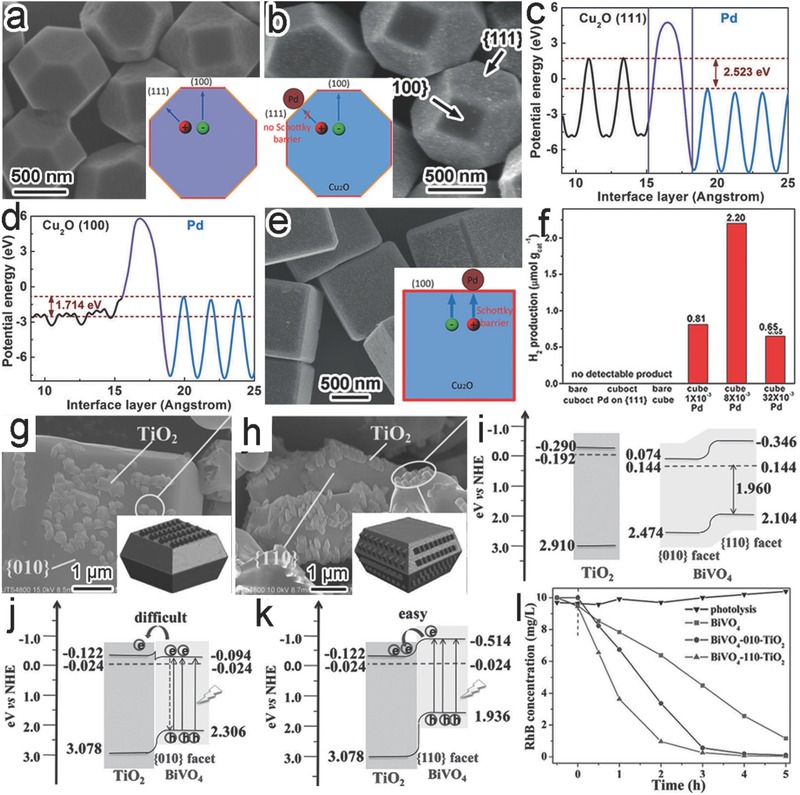
a) SEM image of Cu_2_O cuboctahedrons and schematic illustration (inset) for the charge spatial distribution between the {100} and {111} facet. b) SEM image of Cu_2_O cuboctahedrons with {111} surface decorated with Pd and schematic illustration (inset) for the charge transfer on the Cu_2_O{111}‐Pd interface. c) Potential lineup diagram for Cu_2_O{111}‐Pd interface obtained from first‐principles simulations. d) Potential lineup diagram for Cu_2_O{100}‐Pd interface obtained from first‐principles simulations. e) SEM image of {100}‐faceted Cu_2_O cubes decorated with Pd and schematic illustration (inset) for the charge transfer on the Cu_2_O{100}‐Pd interface. f) Photocatalytic hydrogen production from pure water by Cu_2_O‐based photocatalysts. Reproduced with permission.^21^ g,h) SEM images and schematic illustration (inset) of (g) BiVO_4_‐010‐TiO_2_ and (h) BiVO_4_‐110‐TiO_2_ heterojunctions. i–k) Relative energy band levels of (i) TiO_2_, BiVO_4_{010} facet, and BiVO_4_{110} facet, and (j,k) two types of BiVO_4_‐TiO_2_ heterojunctions with different contact facets. l) Photocatalytic activities of BiVO_4_‐based photocatalysts in RhB degradation under visible‐light irradiation. Reproduced with permission.^122^

In the facet‐engineered interface design, the facet‐dependent electronic band structure in semiconductor is another key factor. It causes different energy band alignments on the formed interfaces and influences the efficiency of interfacial charge transfer. For instance, the work function of Cu_2_O{100} (ca. 7.2 eV) is much higher than that of Cu_2_O{111} (ca. 4.8 eV) in Cu_2_O cuboctahedrons,^21^ leading to the accumulation of electrons and holes on the {100} and {111} facet, respectively (**Figure** **15**a). To facilitate charge separation, a Schottky junction may be built between p‐type Cu_2_O and Pd to trap the photogenerated holes. Determined by spatial charge distribution, the Cu_2_O{111} facets with hole accumulation should be an ideal location to form Cu_2_O{111}‐Pd interface for hole trapping (Figure 15b). Unfortunately, the Schottky junction could not be formed at the Cu_2_O{111}‐Pd interface as Cu_2_O{111} has a lower work function than Pd (Figure 15c).^11^ In stark contrast, the higher work function of Cu_2_O{100} than Pd favored the establishment of Schottky barrier (Figure 15d). For this reason, the Pd‐decorated Cu_2_O cubes with Cu_2_O{100}‐Pd interface should be used for hole trapping instead (Figure 15e). As shown in Figure 15f, the hybrid structures between Cu_2_O cubes and Pd nanoparticles showed more prominent H_2_ production in comparison with other Cu_2_O counterparts.

Following a similar mechanism, the BiVO_4_‐TiO_2_ interfaces were designed for the flow of photogenerated electrons from BiVO_4_ to TiO_2_ under visible‐light irradiation.^122^ Selectively growing TiO_2_ on {010} and {110} facet of BiVO_4_, two different BiVO_4_‐TiO_2_ heterojunctions (namely, BiVO_4_‐010‐TiO_2_ and BiVO_4_‐110‐TiO_2_) were formed, respectively (Figure 15g,h). Due to the lower CBM and VBM of BiVO_4_{010} than BiVO_4_{110}, the BiVO_4_{010}‐TiO_2_ interface exhibited a higher interfacial CB energy barrier as compared with BiVO_4_{110}‐TiO_2_ interface (Figure 15i–k). This feature hindered the electron transfer from BiVO_4_{010} to TiO_2_. As a result, the BiVO_4_‐110‐TiO_2_ heterojunction exhibited higher photocatalytic performance in RhB degradation (Figure 15l).

The third factor for facet‐dependent interfacial charge transfer is associated with the correlation of interfacial structural and electronic couplings with facet contacts. Based on this correlation, we have designed a Ag‐BiOCl‐Pd hybrid structure for synergizing the interfacial charge transfer of plasmonic metal‐semiconductor (Ag‐BiOCl) with semiconductor‐metal (BiOCl‐Pd) Schottky junctions.^22^ In a plasmonic metal‐semiconductor interface, the injection of plamsonic hot carriers to semiconductor follows an opposite direction to the charge trapping by metal through the Schottky junction.^123^, ^124^ In our design, two different metal‐semiconductor interfaces were established to circumvent this undesired situation, in which the semiconductor facets and metals were rationally selected for the plasmonic effect and Schottky junction, respectively.

**Figure 16:**
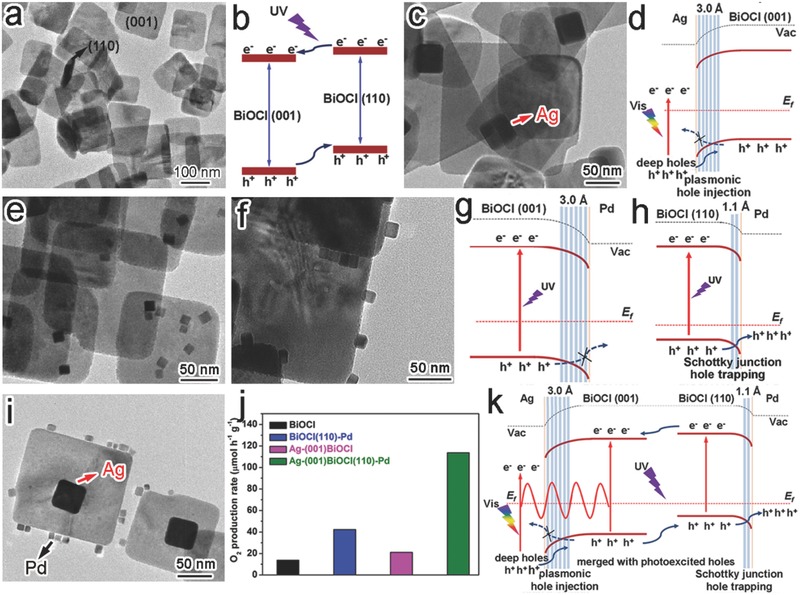
a) TEM image of BiOCl nanoplates. b) Schematic illustration showing the facet‐dependent charge migration in BiOCl. c) TEM image of Ag‐{001}BiOCl. d) Schematic illustration for energy band alignment and hole carrier migration at Ag{100}‐BiOCl{001} interface. e,f) TEM images of (e) BiOCl{001}‐Pd and (f) BiOCl{110}‐Pd. g,h) Schematic illustration for energy band alignment and hole carrier migration at (g) BiOCl{001}‐Pd{100} interface and (h) BiOCl{110}‐Pd{100} interface. i) TEM image of Ag‐{001}BiOCl{110}‐Pd hybrid structure. j) Photocatalytic O_2_ evolution from water by BiOCl‐based materials under full‐spectrum irradiation. k) Schematic illustrating the band alignment and charge flow at two metal‐semiconductor interfaces in Ag‐{001}BiOCl{110}‐Pd under full‐spectrum irradiation. Reproduced with permission.^22^

The design begins with the facet‐dependent charge distribution in p‐type BiOCl nanoplates (**Figure** **16**a). The photogenerated holes and electrons are preferentially accumulated on four side {110} and top/bottom {001} facets, respectively (Figure 16b). Based on this spatial charge separation, two interfaces were designed. The first interface was formed between BiOCl{001} and plasmonic Ag, where the Ag injected hot holes into BiOCl under visible‐light illumination. The other interface was formed between BiOCl{110} and nonplasmonic Pd to establish the Schottky barrier so that the holes can be trapped by the Pd. To prove the rationality of the design, we have investigated the charge transfer of the two interfaces. At the Ag{100}‐{001}BiOCl interface, the plasmonic hot holes in Ag generated by visible light were injected into BiOCl, while the Ag could not effectively trap the holes from the UV‐photoexcited BiOCl through a Schottky junction (Figure 16c). The key to this interface was the thick contact barrier layer (3.0 Å), which disfavored the migration of photoexcited holes from BiOCl{001} to Ag but did not bother the injection of plasmonic hot holes into the VB of BiOCl (Figure 16d). At the other BiOCl{110}‐Pd{100} interface, the interfacial barrier layer was thin enough (1.1 Å) to allow efficient hole trapping through the Schottky junction under UV‐light irradiation (Figure 16e–h). To integrate the two interfaces, Ag‐{001}BiOCl{110}‐Pd hybrid structure was fabricated by selectively depositing Ag and Pd nanocubes on the {001} and {110} facets of BiOCl, respectively (Figure 16i). The synergetic effect of Ag{100}‐{001}BiOCl and BiOCl{110}‐Pd{100} interfaces offerred the highest photocatalytic performance in O_2_ evolution under full‐spectrum irradiation (Figure 16j). In this Ag‐{001}BiOCl{110}‐Pd ternary structure, the two interfaces steered the charge flow through three effects – the plasmonic deep hole injection from Ag through the Ag{100}‐{001}BiOCl interface, the intrinsic facet‐dependent spatial charge separation inside the BiOCl, and the Schottky junction with Pd to extract holes from BiOCl through the BiOCl{110}‐Pd{100} interface (Figure 16k).

**Figure 17:**
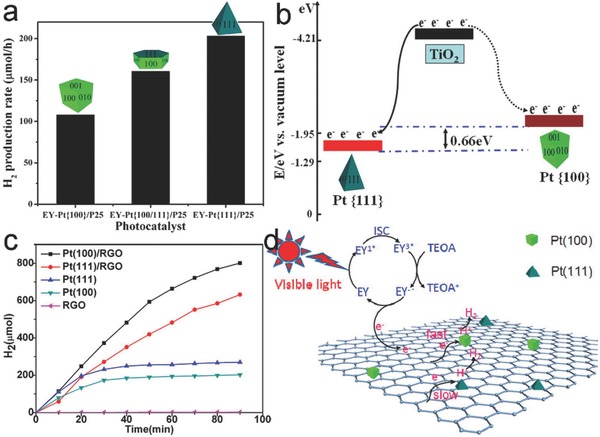
a) Photocatalytic H_2_ evolution rate from triethanolamine (TEOA) aqueous solution on EY‐TiO_2_‐Pt{100}, EY‐TiO_2_‐Pt{100/111}, and EY‐TiO_2_‐Pt{111} under visible‐light irradiation. b) Energy band diagrams for Pt{100} (dotted curve) and Pt{111} facets (solid curve) relative to TiO_2_. Reproduced with permission.^120^ Copyright 2013, American Chemical Society. c) H_2_ evolution from EY‐photosensitized systems catalyzed by RGO, Pt{100}, Pt{111}, Pt{100}/RGO, and Pt{111}/RGO. d) Proposed photocatalytic mechanism for hydrogen evolution over Pt{100}/RGO and Pt{111}/RGO cocatalysts under visible‐light irradiation. Reproduced with permission.^125^ Copyright 2015, American Chemical Society.

Certainly the facet‐engineered interface design is not limited in the selection of semiconductor facets in defining the interface. The facets of other components (e.g., metal) can also be tailored to promote interfacial charge transfer. The metal possesses facet‐dependent work function, altering the interfacial band alignment with semiconductor. For instance, the Eosin Y (EY)‐sensitized Pt/TiO_2_(P25) samples with different exposed facets (e.g., {100}, {100/111}, and {111}) of Pt were employed as photocatalysts for H_2_ evolution under visible‐light irradiation.^120^ The EY‐Pt{111}/TiO_2_ photocatalyst exhibited higher activity than EY‐Pt{100}/TiO_2_ and EY‐Pt{100/111}/TiO_2_ (**Figure** **17**a), as the higher Fermi level of Pt{111} enabled larger difference with the CB of TiO_2_ promoting the electron trapping on Pt{111} (Figure 17b).

Moreover, the interfacial coupling tuned by metal facets may affect the efficiency of charge transfer. In the case of EY‐sensitized Pt/reduced graphene oxide (rGO), different exposed facets of Pt (e.g., Pt{100}/rGO and Pt{111}/rGO) were integrated with the rGO as visible‐light photocatalysts. The photoinduced electrons were transfered to Pt cocatalysts across the rGO‐Pt interface for H_2_ evolution.^125^ The H_2_ evolution activity by Pt{100}/rGO turned out to be substantially higher than that of Pt{111}/rGO, despite the superior performance of bare Pt{111} to Pt{100} (Figure 17c). This performance turnover was caused by the stronger interaction between Pt{100} and rGO, resulting in faster charge transfer at the rGO‐Pt{100} interface (Figure 17d). In general, facet‐engineered design has been carried out for various interfaces of photocatalysts, in efforts to trap charges for redox reactions or extract charges from light‐sensitized components (e.g., plasmonic metal, and semiconductor sensitizer). The comparison of interfacial charge transfer efficiency with different facet contacts in various photocatalytic hybrid materials has been summarized in **Table** **3**.

**Table 3:** Efficiency of interfacial charge transfer with different facet contacts in various photocatalytic hybrid materials as well as their corresponding mechanisms

Photocatalytic hybrid materials	Type of charge transfer	Comparison of efficiency in interfacial charge transfer	Mechanism	Ref.
Pt‐BiVO_4_‐MnO_x_	Charge trapping	Pt‐{010}BiVO_4_{110}‐MnO_x_ > Pt‐{010}/{110}BiVO_4_{010}/{110}‐MnO_x_	Facet‐dependent charge accumulation	20
TiO_2_‐PtO	Charge trapping	TiO_2_{001}‐PtO > TiO_2_{101}‐PtO	Facet‐dependent charge accumulation	121
Cu_2_O‐Pd	Charge trapping	Cu_2_O{100}‐Pd > Cu_2_O{111}‐Pd	Different energy band alignments	21
BiVO_4_‐TiO_2_	Charge injection	BiVO_4_{110}‐TiO_2_ > BiVO_4_{010}‐TiO_2_	Different energy band alignments	122
BiOCl‐Pd	Charge trapping	BiOCl{110}‐Pd{100} > BiOCl{001}‐Pd{100}	Different interfacial couplings	22
TiO_2_‐Pt	Charge trapping	TiO_2_‐Pt{111} > TiO_2_‐Pt{100}	Different energy band alignments	120
Graphene‐Pt	Charge trapping	Graphene‐Pt{100} > graphene‐Pt{111}	Different interfacial couplings	125
TiO_2_‐Pt	Charge trapping	TiO_2_{001}‐Pt > TiO_2_{010}‐Pt	Different interfacial couplings	115
Au‐BiOCl	Hot charge injection	Au‐BiOCl{010} > Au‐BiOCl{001}	Different energy band alignments	42
TiO_2_‐graphene	Charge trapping	TiO_2_{100}‐graphene > TiO_2_{101}‐graphene > TiO_2_{001}‐graphene	Different interfacial couplings	43
Cu_2_WS_4_‐Pt	Charge trapping	Cu_2_WS_4_{101}‐Pt > Cu_2_WS_4_{001}‐Pt	Different energy band alignments	49
PbTiO_3_‐Pt	Charge trapping	PbTiO_3_ positively charged {001}‐Pt > PbTiO_3_ positively/negatively charged {001}‐Pt	Facet‐dependent charge accumulation	52
Cu_2_O‐Pd	Charge trapping	Cu_2_O{100}‐Pd{100} > Cu_2_O{100}‐Pd(without determined facet of Pd)	Different interfacial couplings	58
Au‐Cu_2_O‐Co_3_O_4_	Charge trapping	Au‐{100}Cu_2_O{111}‐Co_3_O_4_ > Au‐{111}Cu_2_O{111}‐Co_3_O_4_	Facet‐dependent charge accumulation	107
TiO_2_‐C_3_N_4_	Charge injection	TiO_2_{101}‐C_3_N_4_ > TiO_2_{001}‐C_3_N_4_	Facet‐dependent charge accumulation	126
Cu_2_O‐graphene	Charge trapping	Cu_2_O{111}‐graphene > Cu_2_O{110}‐graphene > Cu_2_O{100}‐graphene	Different interfacial couplings	127
K_2_La_2_Ti_3_O_10_‐Ni	Charge trapping	K_2_La_2_Ti_3_O_10_{101}‐Ni{111} > K_2_La_2_Ti_3_O_10_{002}‐Ni{111}	Different interfacial couplings	128

## 6 Conclusions and Perspectives

Facet‐engineered surface and interface design of photocatalytic materials represents an interesting and important development direction toward enhanced photocatalytic performance. Thereinto, the facet‐engineered surface design has been carried out for both mono‐component and multi‐component photocatalytic structures, in efforts to enhance surface catalytic reactions by controlling the exposed facets of catalytic components. Three key factors are responsible for the dependence of surface reaction activity on the exposed facets: 1) the adsorption and activation of reactants on surface facets; 2) the surface electronic band structures of various facets determining the redox abilities of photogenerated charge carriers; and 3) accumulation of photogenerated electrons or holes on different facets.

Meanwhile, the facet‐engineered interface design has implemented for the interface between light‐harvesting and catalytic components in multi‐component hybrid structures, which can promote interfacial charge transfer by tailoring the contact facets. The efficiency of interfacial charge transfer is manuevered by contact facets due to three main reasons: 1) the accumulation of photogenerated electrons or holes on the facets that form the interface; 2) the interfacial energy band alignment depending on the surface state structures of different facets; 3) the interfacial structural and electronic couplings relying on the facet contacts.

Based on the fundamental understanding, facet adjustment has been performed for the surface and interface of photocatalytic materials through various synthetic methods. The use of facet‐selective capping agent, kinetic control, thermodynamic control, and anisotropic etching have been developed for tailoring the exposed facets on surface. In comparison, the facet engineering for interface structures is relatively challenging. Thus far, limited success has been made by employing facet‐selective capping agents, facet‐dependent photodeposition, and facet‐dependent selective adsorption. It is anticipated that more efforts will be made toward the interface engineering in the future.

Enabled by the well‐established techniques for surface control, the photocatalytic efficiency for mono‐component semiconductor structures has been significantly improved through either increasing the percentage of the surface facets with high catalytic activity or optimizing the ratio of different facets in synergistic effects. As for multi‐component hybrid structures, the enhancement on photocatalytic performance has been mainly achieved by following the same strategy as bare semiconductors – rationally exposing the component facet with high catalytic activity on surface. The other important factor for hybrid structures – effciency of interfacial charge transfer – has been optimized to some extent by selecting appropriate facets for interface formation; however, it is still a long way to go along this development direction.

Although some encouraging advances have been achieved for the facet‐engineered surface and interface design of photocatalytic materials in the recent years, it still remains a grand challenge to fully exert the functions of facet engineering to photocatalysis. In our opinion, the most challenging task is to control the material interface inside a multiple‐component hybrid structure. This challenge not only originates from the limited synthetic techniques for controlling the interfacial facets, but also requires advanced characterization techniques to probe the charge kinetics at the interface.

In terms of the synthesis, the formation of many interface structures suffers from the large lattice mismatch between components, reducing the interfacial structural and electronic couplings. To overcome the interfacial strain induced by lattice mismatch, non‐epitaxial growth should be a promising approach. In this growth scheme, the nucleation and growth require that the existing component can provide the sites with strong binding to the newly formed one. From the success in literature (e.g., Pd‐Cu_2_O and TiO_2_‐Pd), chemical bondings such as Pd–O can make an important contribution to the non‐epitaxial growth. Another limitation lies on the fact that morphology‐controlling agents are still commonly involved in the existing synthetic methods. The presence of capping agents on component surface will inevitably compromise the quality of interface structures. As for the mechanism research, the characterization techniques at high spatial, spectral and temporal resolutions are highly desirable for probing the interfacial charge kinetics and enabling deeper understanding on the facet‐dependent photocatalytic behaviors. Furthermore, modern theoretical simulations and calculations are also required to raise the level of understanding on the facet effect on photocatalysis and to provide guidance for the facet engineering toward the design of photocatalytic materials. Taken together, the research at the intersection of precise syntheses, advanced characterizations and theoretical simulations can help establish the relationship between facet structure and photocatalytic performance,^11^, ^129^, ^130^ which will in turn formulate well‐designed surface and interface facets for highly efficient photocatalysts.
